# ﻿Characterisation and comparative analysis of mitochondrial genomes of false, yellow, black and blushing morels provide insights on their structure and evolution

**DOI:** 10.3897/imafungus.16.138363

**Published:** 2025-02-21

**Authors:** Gang Tao, Steven Ahrendt, Shingo Miyauchi, XiaoJie Zhu, Hao Peng, Kurt Labutti, Alicia Clum, Richard Hayes, Patrick S. G. Chain, Igor V. Grigoriev, Gregory Bonito, Francis M. Martin

**Affiliations:** 1 College of Eco-Environmental Engineering, Guizhou Minzu University, 550025, Guiyang, China Université de Lorraine Champenoux France; 2 Université de Lorraine, INRAE, UnitéMixte de Recherche Interactions Arbres/Microorganismes, Centre INRAE Grand Est Nancy, 54280 Champenoux, France Guizhou Minzu University Guiyang China; 3 U.S. Department of Energy Joint Genome Institute, Lawrence Berkeley National Laboratory, Berkeley, CA 94720, USA U.S. Department of Energy Joint Genome Institute, Lawrence Berkeley National Laboratory Berkeley United States of America; 4 Okinawa Institute of Science and Technology Graduate University, Onna, Okinawa 904-0495, Japan Okinawa Institute of Science and Technology Graduate University Onna, Okinawa Japan; 5 Los Alamos National Laboratory (LANL), Los Alamos, NM 87545, USA Los Alamos National Laboratory Los Alamos United States of America; 6 Department of Plant and Microbial Biology, University of California Berkeley, Berkeley, CA 94720, USA University of California Berkeley Berkeley United States of America; 7 Plant Soil and Microbial Sciences, Michigan State University, East Lansing, MI 48824, USA Michigan State University East Lansing United States of America

**Keywords:** Genomic synteny, homing endonuclease gene, mitochondrial genome, *Morchella*, protein-coding gene

## Abstract

*Morchella* species have considerable significance in terrestrial ecosystems, exhibiting a range of ecological lifestyles along the saprotrophism-to-symbiosis continuum. However, the mitochondrial genomes of these ascomycetous fungi have not been thoroughly studied, thereby impeding a comprehensive understanding of their genetic makeup and ecological role. In this study, we analysed the mitogenomes of 30 *Morchellaceae* species, including yellow, black, blushing and false morels. These mitogenomes are either circular or linear DNA molecules with lengths ranging from 217 to 565 kbp and GC content ranging from 38% to 48%. Fifteen core protein-coding genes, 28–37 *tRNA* genes and 3–8 *rRNA* genes were identified in these *Morchellaceae* mitogenomes. The gene order demonstrated a high level of conservation, with the *cox1* gene consistently positioned adjacent to the *rnS* gene and *cob* gene flanked by *apt* genes. Some exceptions were observed, such as the rearrangement of *atp6* and *rps3* in *Morchellaimportuna* and the reversed order of *atp6* and *atp8* in certain morel mitogenomes. However, the arrangement of the *tRNA* genes remains conserved. We additionally investigated the distribution and phylogeny of homing endonuclease genes (HEGs) of the LAGLIDADG (LAGs) and GIY-YIG (GIYs) families. A total of 925 LAG and GIY sequences were detected, with individual species containing 19–48HEGs. These HEGs were primarily located in the *cox1*, *cob*, *cox2* and *nad5* introns and their presence and distribution displayed significant diversity amongst morel species. These elements significantly contribute to shaping their mitogenome diversity. Overall, this study provides novel insights into the phylogeny and evolution of the *Morchellaceae*.

## ﻿Introduction

Mitochondria are semi-autonomous organelles that play a pivotal role in fungal respiratory metabolism and energy production (Burger et al. 2003; Wai and Langer 2016). They also play a role in regulating ion balance and intermediary metabolic processes and have been linked to cell death and virulence of pathogenic fungi (Verma et al. 2018; Black et al. 2021). Each mitochondrion has a distinct mitogenome. In fungi, mtDNA exists in either a circular or linear configuration and multiple copies may be present in a single cell. Although smaller than that of the nuclear genome, mtDNA exhibits significant variability (Burger et al. 2003; Aguileta et al. 2014).

The evolutionary history of fungal mitogenomes warrants further investigation, particularly considering their high variability in size and gene arrangement. Previous studies have reported diverse genome sizes, ranging from 11.2 kb (*Hanseniasporapseudoguilliermondii*) to 332.2 kb (*Golovinomycescichoracearum*), which have primarily been attributed to differences in intergenic regions, intron number and intronic ORFs (Bullerwell et al. 2003; Sethuraman et al. 2009; Joardar et al. 2012; Deng et al. 2018). Variations in gene arrangements make them valuable tools for examining fungal evolution (Bullerwell and Lang 2005; Megarioti and Kouvelis 2020; Christinaki et al. 2022). Despite these variations, most fungal mitogenomes contain a conserved set of 15 core protein-coding genes (PCGs) dedicated to energy metabolism (Li et al. 2021), a single *rps3* gene responsible for transcriptional regulation (Korovesi et al. 2018), 22-36 *tRNA* genes and two *rRNA* genes (Barroso et al. 1995; Formighieri et al. 2008).

The presence of repeated DNA sequences, such as introns that display self-splicing and insertion endonuclease activity, can significantly impact the structural dynamics of fungal mitochondrial genomes. This can result in variations in gene order, dispersion of repetitive elements and introduction of new genes through horizontal gene transfer (HGT) (Ferandon et al. 1995; Ferandon et al. 2010). Additionally, the distribution of transfer RNAs (*tRNAs*) can influence gene arrangement, with *tRNAs* being capable of editing, excising and integrating into different locations within the genome, allowing for their participation in HGT events, as reported by Tuller et al. (2011). Analysis of *tRNA* placement in fungal mitochondrial genomes is a valuable tool for investigating fungal evolution and extracting phylogenetic information, as highlighted by Cedergren and Lang (1985).

Homing endonuclease genes (HEGs) are commonly located within the introns of fungal mitogenomes and are distributed amongst the *cox*, *cob*, *nad* and *rRNA* genes (Novikova and Belfort 2017; Gomes et al. 2018; Li et al. 2018; Li et al. 2020). These genes play a crucial role in the high diversity observed and can modify the organisation and size of the mitogenome (Ferandon 2010; Hafez and Hausner 2012; Ferandon and Barroso 2013). In fungi, HEGs of three families are found, but only the LAGLIDADG and GIY-YIG genes are unique to the fungal mitogenomes (Hafez and Hausner 2012; Li et al. 2019a; Wang et al. 2020). These genes exhibit a wide range of sizes and subtypes, resulting in variable intergenic regions and inclusion of introns within their sequences (Belfort et al. 2007; Megarioti et al. 2020). HEGs possess independent open reading frames (ORFs) and employ unique self-splicing mechanisms that play a crucial role in the co-evolution of introns and HEGs (Belfort and Roberts 1997; Chevalier and Stoddard 2001; Megarioti et al. 2020). Additionally, some HEGs may serve supplementary biological functions, such as maturase activity and transcriptional repression (Stoddard 2014). The presence of HEGs significantly contributes to the diversification and evolution of fungal mitogenomes.

Morels belong to the *Morchella* genus (*AscomycotaMorchellaceae*). Although only 59 are formally recognised as valid Latin binomials (Machuca et al. 2021; Clowez et al. 2022; Loizides et al. 2022), over 358 species have been documented in the Index Fungorum database (http://www.indexfungorum.org/Names/Names.asp). The fruiting bodies of several morel species are known for their high nutritional value and diverse medicinal activities, including anti-inflammatory, anti-oxidative, anti-viral and anti-tumour effects (Liu et al. 2016; He et al. 2017; Liu et al. 2018). Additionally, these species exhibit a wide range of ecological lifestyles along the saprotrophism-to-symbiosis continuum (O’Donnell et al. 2011). Although most *Morchella* species, including those cultivated for human consumption, are generally regarded as soil or litter decomposers (Hobbie et al. 2016; Benucci et al. 2019), some species are thought to establish associations with plant roots (Baynes et al. 2012) or exhibit a propensity for bacterial interactions (Pion et al. 2013). The structure and activity of the mitogenome can differ, based on the ecology of morels. Regrettably, only a few morel mitogenomes have been extensively examined (Liu et al. 2020a, 2020b). This scarcity impedes the development of a comprehensive understanding of genetic traits and various ecological functions.

In this study, we sequenced, annotated and characterised the mitogenomes of 30 *Morchellaceae* species that encompass a range of phylogenetic lineages (false morels, blushingmorels, black morels and yellow morels) and ecological types. Our objectives were: to elucidate the genetic characteristics of these mitogenomes; compare the phylogeny based on the core PCGs with the nuclear genome phylogeny; analyse the composition, distribution and synteny of PCGs and HEGs; and gain insight into the phylogenetic features and potential impact of lifestyle. This study constitutes the first comprehensive investigation of the mitogenomes of *Morchellaceae* and provides valuable information on the phylogeny, genomics and ecological roles of this significant fungal group.

## ﻿Materials and methods

### ﻿Samples collection and mitogenome sequencing

Thirty fungal strains, including 28 *Morchella* species and two false morels (*Disciotisvenosa* NRRL24433 and *Verpaconica* TJ0815), were collected from specific locations (Table 1). The *Morchella* species were primarily categorised into three groups: black, yellow and blushing morels, which were supported by molecular phylogenetic data and described as the *Esculenta*, *Elata* and *Rufobrunnea* clades (O’Donnell et al. 2011) and the *Morchellaceae* tree on the basis of their nuclear genomes constructed by the Joint Genome Institute (JGI) MycoCosm (Tree-*Morchellaceae* (https://mycocosm.jgi.doe.gov/mycocosm/species-tree/tree;Kglla0?organism=morchellaceae). The specimens were subsequently deposited as vouchers at the collection centre.

**Table 1.** General statistics and taxonomic information for the 30 species. **mtDNA size**, mitochondrial genome size; **No. scf**, the number of scaffold; **1. No. t RNA**: number of tRNA genes; **2. No. LAGs**, the number of LAGLIDADG homing endonuclease genes; **3. No. GIYs**, the number of GIY-YIG homing endonuclease genes; **4. No. rnL**, rRNA large subunit genes; **5. No. rnS**, rRNA small subunit genes; **6. No. HGs**, hypothetical protein genes.

Portal ID	Species name	mtDNA size (bp)	No. scf	G+C%	Topology	No. tRNA^1^	No. LAGs^2^ /GIYs^3^	No. rnL ^4^	No. rnS ^5^	No. HGs ^6^
Morcon1	*Morchellaconifericola* Mel32	263666	1	40.72	circular	31	31	2	2	23
Morsep1	*Morchellaseptentrionalis* NRRL54509	264912	1	40.65	circular	31	33	2	1	17
Morarb1	*Morchellaarbutiphila* PhC291	261722	1	40.68	circular	31	31	2	2	33
Morbru1	*Morchellabrunnea* NRRL20869	264219	1	40.88	circular	31	29	2	1	19
Morhis1	*Morchellahispaniolensis* Mel18	272680	1	40.54	circular	31	34	2	2	18
MorM21481-1	*Morchella* Mel-23	254381	1	40.63	linear	28	29	2	2	25
Mordel1	*Morchelladeliciosa* PhC191	304819	1	40.67	circular	31	38	**5**	3	**14**
Morkaki1	*Morchellakakiicolor* PhC280	339294	1	42.2	linear	33	31	1	2	26
Morsem1	*Morchella* sp. SEM	339433	1	42.2	circular	33	34	1	2	33
Morgal1	*Morchella* sp. GAL	340119	1	42.33	circular	32	33	1	2	**3**
Mordis1	*Morchella* sp. DIS	339404	1	42.2	circular	33	34	1	2	32
Mordun1	*Morchelladunalii* PhC240	339982	1	42.41	circular	31	32	3	2	**45**
Morimp1	*Morchellaimportuna* SCYDJ1-A1	274206	2	38.22	linear	30	24 / (**2**)	2	2	**1**
Morexi1	*Morchellaeximia* NRRL26621	305324	1	41.46	circular	32	40 / (**1**)	1	3	**3**
Morexim1	*Morchellaeximia* DOB1602	282169	1	41.85	circular	32	28 / (**1**)	1	2	33
Morpop1	*Morchellapopuliphila* NRRL22315	370799	1	40.18	linear	32	48	1	3	**5**
Morpun1	*Morchellapunctipes* GB769	351597	1	40.57	linear	30	43/ (**1**)	**1**	2	13
Mortrid1	*Morchellatridentina* NRRL54570	303561	1	40.06	circular	31	41	3	2	26
MorM1934m1-1	*Morchellafluvialis*	558743	1	46.81	linear	34	20	1	2	47
MorvulMes17-1	*Morchellavulgaris* Mes-17	561093	2	46.91	linear	32	21	2	2	**108**
Morpra1	*Morchellaprava* Mes7	565090	1	46.79	circular	32	22	2	2	56
Morper1	*Morchellaperuviana* NRRL66754	554401	1	46.1	circular	32	29	1	2	68
Morulm1	*Morchellaulmaria* NRRL36825	558995	1	46.92	linear	33	20 / (**1**)	3	2	45
Morame1	*Morchellaamericana* PhC192	555871	1	48.33	linear	32	19	2	2	36
Mordim1	*Morchelladiminutiva* Mes2	471506	1	46.16	circular	33	22	2	1	30
Morpal1	*Morchellasteppicola*	351320	1	41.23	circular	31	34	2	1	18
Morana1	*Morchellaanatolica* PhC233	475320	1	42.26	circular	32	34		3	63
Morruf1	*Morchellarufobrunnea* NRRL28464	446548	1	40.92	circular	32	30	2	3	51
Disven1	*Disciotisvenosa* NRRL24433	265955	1	37.45	circular	32	30	3	2	36
Vercon1	*Verpaconica* TJ0815	217659	1	38.48	circular	37	25	2	2	47

### ﻿Mitogenome sequencing, assembly and annotation

The draft genomes of the *Morchellaceae* species were generated at the DOE Joint Genome Institute (JGI) using PacBio technology. A PacBio Multiplexed >10 kb w/ Blue Pippin Size Selection library was constructed and sequenced using SEQUELIIe, which generated >500,000 reads, totalling > 6 Gb. CCS data were filtered with the JGI QC pipeline to remove artefacts. Mitochondrial genomes were assembled separately from CCS reads as follows: CCS reads likely to belong to organelles were separated from nuclear genome reads using coverage and GC filtering. A maximum coverage cutoff of (1.5 * kmer coverage peak) and a maximum GC fraction of 0.40 was used to exclude nuclear content using BBTools (B. Bushnell: BBTools software package, http://sourceforge.net/projects/bbmap) version 38.79 [kmer count exact.sh default; bbnorm.sh pigz passes = 1 bits = 16 target = 9999999 min = 162; bbduk.sh maxgc = 0.4]. Initial mitochondrial assemblies were produced using Flye version 2.9-b1768 [-g 100 —asm-coverage 100—pacbio-hifi] (Kolmogorov et al. 2019). Genes were predicted in the assembly using Prodigal software version 2.6.3 [-p meta] (Hyatt et al. 2010) and were searched against an in-house curated database of mitochondrial HMMs using HMMER hmmsearch version 3.1b2 [-domtblout] (Mistry et al. 2013). Contigs with putative mitochondrial genes were predicted using ribosomal loci masked with BB tools [bbduk.sh k=25 mm=f kmask=N] and an in-house curated database of common eukaryotic nuclear ribosomal sequences. The masked contigs were used to recruit additional CCS reads using BB tools [k=25 mm=f mkf=0.03 ordered ow]. The resulting reads were assembled with flye [-g 100k—asm-coverage 100—pacbio–hifi]. Additional iterations of read recruitment and assembly were performed. Contigs<1 kb were excluded to produce the final assembly, which was then used to filter the CCS reads to produce non-organelle CCS and polished with two rounds of RACON version 1.4.13 [-u-t 36] (Vaser et al. 2017). The mitochondria-filtered CCS reads were then assembled using Flye version 2.9-b1768 [-t 32—pacbio-hifi] and subsequently polished with two rounds of RACON version 1.4.13 racon [-u-t 36]. Mitogenome assemblies were annotated using a workflow developed at JGI (Haridas et al. 2018).

### ﻿Analyses of protein-coding genes(PCGs)

We analysed the GC content and AT/GC skew for PCGs and the entire genomes of 30 *Morchellaceae* species. Mitogenome strand asymmetry was assessed using the following formula: AT skew = [A − T]/[A + T] and GC skew = [G − C]/[G + C] (Li et al. 2021). This process was carried out using the R packages rtracklayer and seqinr and custom R scripts (Charif and Lobry 2007; Lawrence et al. 2009).

### ﻿Phylogenetic analysis

A phylogenetic tree of the *Morchellaceae* species was constructed using concatenated 15 PCG sequences using the OrthoFinder algorithm (Emms and Kely 2019). Aligned orthologous protein sequences were obtained using MAFFT (Katoh et al. 2013) and concatenated using trimAl (Capella-Gutiérrez et al. 2009). Subsequently, a Maximum Likelihood (ML) tree was inferred using the Blosum 62+F+R3 model with 1000 bootstrap replicates using the IQtree algorithm (Minh et al. 2020). Phylogenetic trees of LAGLIDADG genes were constructed using the ML method, based on the amino acid sequences of LAGLIDAG homing endonucleases from individual morels.Trees of the *atp6* and *atp8* genes were constructed using nucleotide sequences with FastTree (Price et al. 2010).

### ﻿Mitogenome synteny and genomic feature association analysis

We analysed the genome statistics and utilised the Synteny-Governed Overview pipeline (SynGO; Hage et al. 2021) to identify the syntenic regions amongst the 30 fungal species. Genomic information from MycoCosm was combined and visualised using the Visually Integrated Numerous Genres of Omics pipeline (VINGO; Looney et al. (2022)). We examined statistically significant variables in genomic features using Permutational Multivariate Analysis of Variance (PERMANOVA). The percentage of variance (R2) contributing to the genomic data was estimated for variables including ecological groups, the size of genomes with genes and phylogenetic distances. The detailed procedures have been previously described (Miyauchi et al. 2020). We tested the differences amongst various groups using the Kruskal-Wallis test with the post hoc Dunn test and the R package DescTools (Signorell et al. 2020). We evaluated the associations between the genomic features. A phylogenetic tree was constructed using the R package ape (Pradis and Schliep 2019). The tree and genomic data were combined using the R package phylobase (Hackathon et al. 2024). Principal components, considering phylogenetic distances, were calculated using the R package adephylo (Jombart and Dray 2010). The generated output files were combined and visualised usinga Proteomic Information Navigated Genomic Outlook (PRINGO; Miyauchi et al. (2020)). Pearson correlation coefficients of genes and genomes were calculated from the size of the genomes with genes using the R basic function *cor.* The results were visualised with custom R scripts using ggplot2 (Wickham 2016).

### ﻿Homing endonuclease gene (HEG) Distribution

The LAGLIDADG and GIY-YIG sequences were located within the PCGs using Artemis Software (version 18.2.0, http://sanger-pathogens.github.io/Artemis/Artemis/), as presented in Table 2. Based on the annotated morel and false-morel mitochondrial genomes, each HEG in the mitochondrial genome was identified by reading the GB format files using the Artemis software. Subsequently, the amino acid sequences of these genes were accessed using the View toolbar in the software and downloaded as FASTA files. The amino acid sequences of the HEGs were then aligned (http://www.ebi.ac.uk/Tools/msa/clustalo/) and classified, based on their integration into core protein-coding genes (e.g., *cob*, *cox* and *nad*) and other regions or non-coding areas within the mitochondrial genome. Comparative analyses were conducted to detect highly conserved amino acid sequences in the mitochondrial genomes of the different strains, as shown in Fig. 6 and Suppl. material 6.

**Table 2.** The LAGLIDADG and GIY-YIG genes/introns occurring within the core genes among the 30 mitogenomes.

Core gene	*cox1*	*nad4*	*nad3*	*cob*	*nad1*	*nad2*	*atp9*	*rps3*	*rnl*	*nad5*	*nad4L*	*cox2*	*cox3*	*rns*	Total gene numbers of LAG or GIY **
Portal ID															
**Morcon1**	12/8	/1		8/4	/2	/1	/1			3/7		2/2	/4		**31/30**
**Morsep1**	12/8	/1		1/2	/2	/1				4/7	1	3/3	/4		**33/28**
**Morarb1**	11/8	/1		8/4	/2	/1	/1			3/7	1	2/2	/4		**31/30**
**Morbru1**	11/8	/1		7/4	/2	/1				3/7		2/2	/4		**29/29**
**Morhis1**	9/7	/1		6/5	1/3	/1				6/8	1	3/3	1/4		**34/32**
**MorM21481-1**	11/8	/1		8/4	/2	/1				3/7	1	2/2	/4		**29/29**
**Mordel1**	10/7	/1		6/4	/2	/1	/1			5/7		4/4	/4		**38/31**
**Morkaki1**	11/8	/1	1	7/5	1/3	/1				1/7		1/2	/4		**31/31**
**Morsem1**	12/8	/1	1	7/5	1/3	/1				1/7		1/2	/4		**34/31**
**Morgal1**	12/8	/1	1	7/4	1/3	/1				1/7		1/2	/4		**33/30**
**Mordis1**	12/8	/1	1	7/5	1/3	/1				1/7		1/2	/4		**34/31**
**Mordun1**	12/8	/1	1	7/5	1/3	/1				1/7		2/3	/4		**32/32**
**Morimp1**	10 (1 GIY) /9	/1		5 (*1 rps3) /3	/2	/3		1		/5		3/3	/4		**26** (**2 GIYs**) /**30**
**Morexi1**	12/9	1/1	1	8/4	1/3	/1				/6	1	1/2	/4	1	**41** (**1 GIY**) /**30**
**Morexim1**	10/9		1	8/4	/2	/1				/6	1	1/2	/4		**29** (**1 GIY**) /**27**
**Morpop1**	21/8	1/1		5/4	/2	/1				5/6		2/2	/4	1	**48/28**
**Morpun1**	17/8	1/1		5/4		/1				4/7		2/3	1/4		**44** (**1 GIY**) /**28**
**Mortrid1**	10/9	/1		8/4	1/3	/1	/1			3/5	1	3/2	/4	1	**41/30**
**MorM1934m1-1**	7/8	/1		2/4	/2	/2	/1			4/9	1	1/3	/4		**20/34**
**MorvulMes17-1**	7/8	/1		2/4	/2	/2				3/9		1/3	/4		**21 /33**
**Morpra1**	7/7	/1		2/4	/2	/2	/2			3/9		1/4	/4		**22/35**
**Morper1**	8/8	/1		3/4	/2	/2	/2			3/9		2/4	/4		**29/36**
**Morulm1**	8/8	/1		3/4	/2	/2	/1			2/8		1/3	/4		**21** (**1 GIY**) /**33**
**Morame1**	6/7	/1		1/4	/2	/2	/2			3/9		1/3	/4		**19/34**
**Mordim1**	6/8	/1		3/3	/2	/2			1	3/9	1	1/3	1/4		**22/32**
**Morpal1**	9/9	/1		11/4	/2	/1				1/4	1	1/3	/4	1	**34/28**
**Morana1**	12/9	/1		1/2	/3	/2	/1			6/9		2/5	1/3		**34/35**
**Morruf1**	9/11	/1		1/2	1/3	/2	/2			7/9		1/4	1/3	1	**30/37**
**Disven1**	7/8			7/3	/1					3/5	1	/2	/3		**30/22**
**Vercon1**	5/4			1/2	/2					/2		1/3	2/4		**25/17**

* means the overlap between *cob* and *rps3*; ** means LAGLIDADG or GIY-YIG.

### ﻿Abbreviations

**Mitogenome** Mitochondrial genome

**HEG** Homing endonuclease gene

**HGT** Horizontal gene transfer

**ORF** Open reading frame

**ML** Maximum Likelihood

**PCG** Protein-coding gene

***atp*** ATP synthase

***cox*** cytochrome c oxidase

***cob*** cytochrome b-coding gene

**NADH** Nicotinamide adenine dinucleotide

***rps*** ribosomal protein

## ﻿Results

### ﻿Mitogenome features and *Morchellaceae* phylogeny

**Figure 1.**
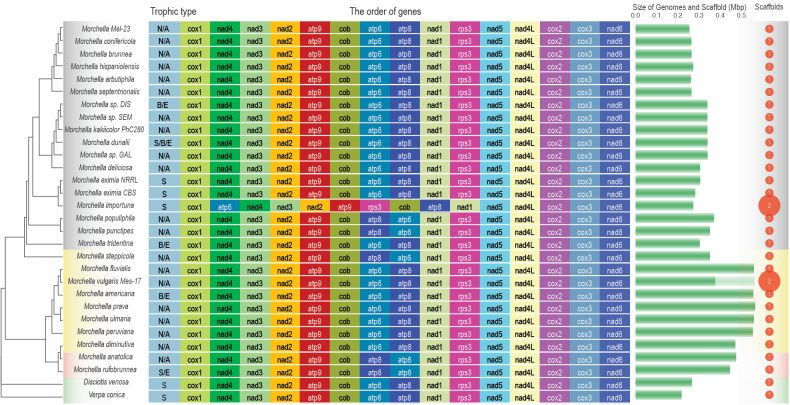
Overview of 30 mitogenomes. General genomic features includes 30 *Morchellaceae* species in the evolutionary order according to the maximum likelihood tree (see Methods). The trophic type indicates N/A: no data. B/E: Biotrophic/endophytic. S/B/E: Saprotrophic/Biotrophic/Endophytic. The order of 15 protein-coding genes (PCGs), and the size of genomes with the number of scaffolds. Phenotypic groups are colour-coded. Grey: Black morels. Yellow: Yellow morels. Red: Blushing morels. Green: False morels. Green gradient bars show the size of the scaffolds and total size of the genomes. The bubbles beside the graph indicate the number of scaffolds in the genome assembly. Note that the gene positions were adjusted in a circular way to facilitate visible relative comparisons. See the linear version of detailed syntenic comparisons (Suppl. material 7: figs S1, S2, S3). See phylogenetic trees of *atp6* and *atp8* genes (Suppl. material 7: fig. S4).

**Figure 2.**
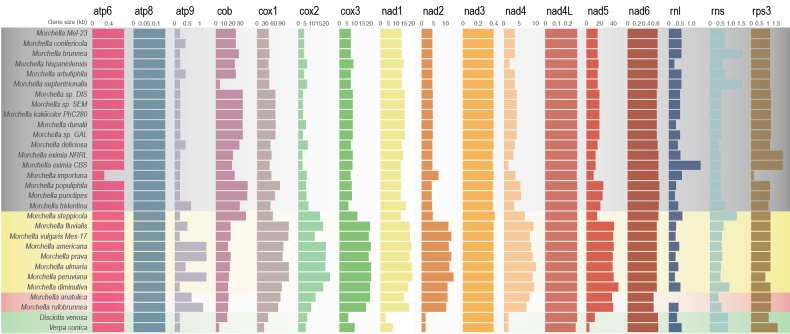
The length of mitogenomic core genes. The core genes are abbreviated. *atp*: ATP synthase. *cob*: apocytochrome b; *cox*: cytochrome c oxidase. *nad*: NADH dehydrogenase. *rnl*: Large subunit *rRNA*. *rns*: small subunit *rRNA*. *rps*: ribosomal protein. The species are in the evolutionary order based on a multi-gene maximum likelihood tree constructed (see Methods). Phenotypic groups are colour-coded. Grey: Black morels. Yellow: Yellow morels. Red: Blushing morels. Green: False morels.

**Figure 3.**
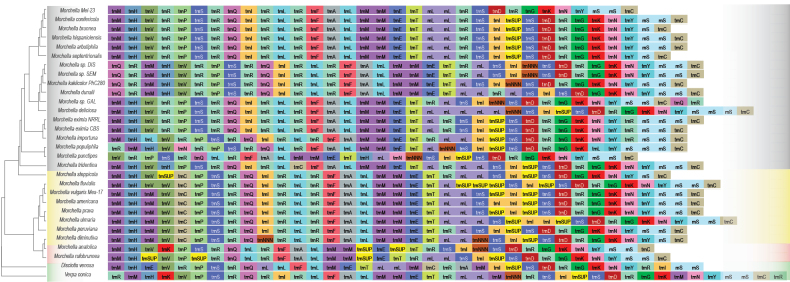
The gene order of *tRNA* and *rRNA* in the mitogenomes. The species are in the evolutionary order based on a multi-gene maximum likelihood tree constructed (see Methods). Phenotypic groups are colour-coded. Grey: Black morels. Yellow: Yellow morels. Red: Blushing morels. Green: False morels. See details (Suppl. material 1).

Mitochondrial genomes of 30 *Morchellaceae* species were sequenced and annotated. The selected species included false morels, blushing and black and yellow morels, representing distinct ecological lifestyles including soil/litter decomposers, endophytes and Y-mycorrhizal species (Buscot 1992; Dahlstrom et al. 2000).The mitogenomes of *Morchellaceae* were predominantly circular, although some species exhibited a linear arrangement (Table 1). The assemblies comprised a single scaffold with the exception of *M.importuna* and *Morchellavulgaris* Mes-17 which have two scaffolds (Table 1 and Fig. 1). The mitogenome size ranged from 254,381 bp in *Morchella* Mel-23to 565,090 bp in *M.prava* Mes7, with an average size of 377,541 bp (Table 1; Fig. 1; Suppl. material 7: figs S1–S3). The GC content ranged from 38.22% in *M.importuna* SCYDJ1-A1 to 48.33% in *M.americana*PhC192, with the highest GC content (48.33%) observed in *M.americana* PhC192 (Table 1). Each of the 28 morel mitogenomes contained two types of *rRNA* genes, one small subunit ribosomal *RNA* gene (*rnS*) and one large subunit ribosomal *RNA* gene (*rnL*), with the exception of *M.anatolica* PhC233, which had three *rnS* genes and a missed *rnL* gene (Table 1, Fig. 2). Furthermore, the mitochondrial genomes encode 28–37 *tRNA* genes for 20 standard amino acids (Table 1, Fig. 3).

Maximum Likelihood phylogenetic analysis was performed using concatenated amino acid sequences from the 15 PCGs (Fig. 1). The resulting tree displayed a high degree of congruence with previous nuclear genome-based phylogenetic topologies (O’Donnell et al. 2011) and the *Morchellaceae* phylogeny, based on nuclear genomes constructed by MycoCosm using single-copy conserved nuclear protein genes (https://mycocosm.jgi.doe.gov/mycocosm/species-tree/tree;vtOK-f?organism=morch-ellaceae). The species tree confirmed that true morels were divided into three main clades corresponding to known phenotypic groups, with the exception of *M.parazonii*, which formed an outgroup to the black morel group (Fig. 1). Notably, blushing and yellow morels were grouped within the same cluster.

### ﻿The base composition of protein-coding genes (PCGs)

PCGs, *tRNA* genes and *rRNA* genes were annotated for the 30 *Morchellaceae* mitogenomes. Fifteen core PCGs were detected: *atp6*, *atp8*, *atp9*, *cob*, *cox1*, *cox2*, *cox3*, *nad1*, *nad2*, *nad3*, *nad4*, *nad4L*, *nad5*, *nad6* and *rps3*. Additionally, a single *rps3* gene, involved in transcriptional regulation, was identified (Fig. 2). The mitogenome sizes of the yellow morels weresignificantly larger than those of the black and false morels (Fig. 1; Suppl. material 7: fig. S5; FDR adj. p < 0.05). Trends in mitogenome size were explained by the phylogenomic distances of the species (Suppl. material 7: fig. S6; R squared of PC1-3 = 0.84; p < 0.05), suggesting that closely-related species have similar-sized genomes.

At the gene level, several PCGs in yellow and blushing morels were larger than their orthologs in black morels, accounting for the mitogenome enlargement observed in these groups (Fig. 2; Suppl. material 7: fig. S5). Notably, these genes included those encoding cytochrome c oxidase (e.g., *cox1*, *cox2* and *cox3*) and NADH dehydrogenase subunits (e.g., *nad1*, *nad2*, *nad4* and *nad5*), which are highly correlated with genome size (Suppl. material 7: fig. S7; Pearson coefficient > 0.7, p < 0.05). In contrast, the cytochrome b-coding gene (*cob*) of black morels was significantly larger than that of yellow morels (Suppl. material 7: fig. S5; FDR adj. p < 0.05). In addition, the size of some genes (e.g., *cob*, *cox* and *nad*) was significantly associated with species relatedness (Suppl. material 7: fig. S6; R squared of PC1 and PC2 > 0.6; P < 0.05).

**Figure 4.**
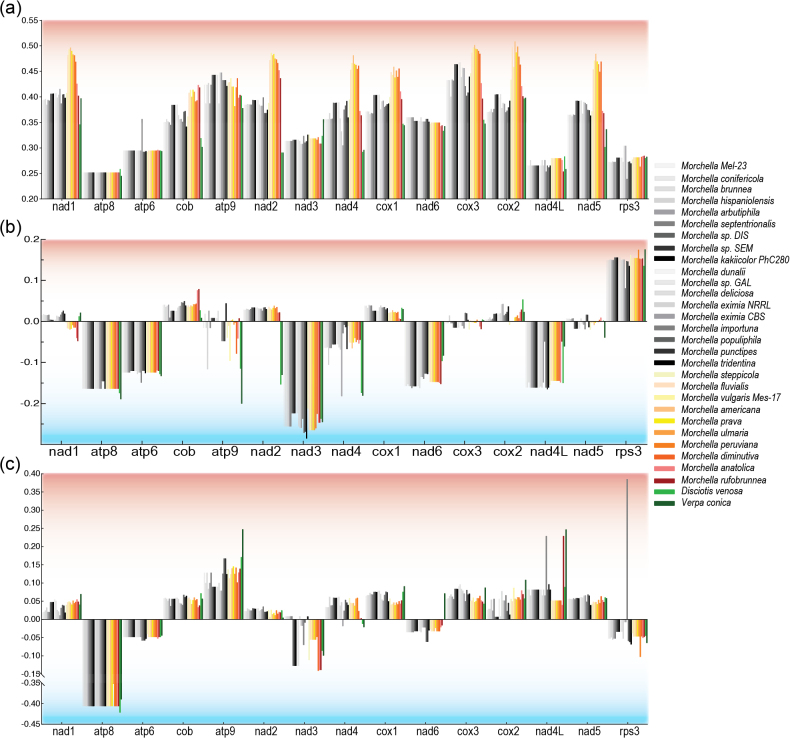
The GC content in the mitogenomes **a** GC content of each of 15 PCGs among 30 fungal mitogenomes **b** AT skew **c** GC skew. Phenotypic groups are colour-coded. Grey: Black morels. Yellow/Orange: Yellow morels. Red: Blushing morels. Green: False morels. See details (Suppl. material 2).

The GC content ranged from 37.45% to 48.33%. *Morchellaamericana* (Morame1) had the highest GC content. Yellow morels displayed a higher GC content than black morels and false morels had the lowest GC content. Upon closer examination, the average GC content of each PCG in the 30 fungi samples varied from 25.17% for *atp8* to 44.42% for *cox3*. Notably, *nad1*, *nad2*, *nad4* and *cob* showed higher GC contents in yellow morels than in black morels (Fig. 4a; Suppl. material 2). The variation in GC content of PCGs was partially explained by the groups (Suppl. material 7: fig. S6). There was a tendency for yellow morels to show significantly higher GC content than black morels (Suppl. material 7: fig. S5). The GC content of *cox1*, *cox2*, *nad1*, *nad2*, *nad4*, *nad5* and *cob* was highly positively correlated with the genome size (p < 0.05; Suppl. material 2).

A clear pattern of positive AT skew (0.016–0.15) was observed for *cox1*, *cox2*, *nad2*, *cob*, *atp6*, *atp8*, *nad3*, *nad4*, *nad6* and *nad4L*, indicating a higher frequency of A than that of T in the forward strand. Conversely, *atp6*, *atp8*, *nad3*, *nad6* and *rps3* showed a negative AT skew, ranging from −0.065 to −0.25. Additionally, all PCGs, except *nad1*, *atp9*, *cox3* and *nad5*, exhibited positive or negative AT skew for each PCG amongst the different models. Furthermore, all PCGs, except for *atp8*, *atp6*, *nad3*, *nad6* and *rps3*, displayed a positive GC skew ranging from 0.022 to 0.122, indicating a higher frequency of G than C in the forward strand. *M.importuna rps3* exhibited a positive GC skew with a value of 0.385 (Fig. 4).

### ﻿PCG microsynteny

We assessed mitogenome gene synteny with a specific focus on the spatial distribution of PCGs across black, yellow and blushing morels (Fig. 1; Suppl. material 7: figs S2, S3). The relative positions and orders of the 15 PCGs were highly conserved within the *Morchellaceae* family. Most PCGs, including *cox1*, *rnS*, *cob* and *apt*, displayed remarkable conservation across all morel clades, signifying robust evolutionary stability. Nevertheless, distinctive differences in gene order were observed, such as the rearrangement of *atp6* and *rps3* in *M.importuna* (Morimp1) and the reversed order of *atp6* and *atp8* in *M.populiphila* (Morpop1), *M.punctipes* (Morpun1), *M.steppicola* (Morpal1), *M.anatolica* (Morana1) and *M.rufobrunnea* (Morruf1) (Fig. 1). Furthermore, the genomic structure of *Morchellavulgaris* Mes-17, a yellow morel, was truncated because of its division into two scaffolds (Fig. 1). Consequently, only the larger scaffold was considered in synteny analysis (Suppl. material 7: fig. S3a). Notably, the genomic arrangement of *M.anatolica* and *M.rufobrunnea* significantly deviated amongst blushing morels, with the exception of specific PCGs, such as *nad4* (Suppl. material 7: fig. S3b), which is likely attributable to disparities in the sequencing starting points and directions.

Comparisons of *atp6* and *atp8* amongst the 30 morels revealed distinct patterns (Suppl. material 7: fig. S4). The *atp6* gene showed that *M.populiphila* (Morpop1) and *M.punctipes* (Morpun1) were outgroups of the black morel group. Intriguingly, *M.importuna* (Morimp1) was distant from black morels and grouped instead with false morels. Meanwhile, the *atp8* gene exhibited mostly identical sequences amongst the fungi, except for some species including *D.venosa*, *V.conica* (Disven1; Vercon1; false morels), *M.populiphila*, *M.punctipes* (Morpop1; Morpun1; black morels) and *M.ulmaria* (Morulm1; yellow morel).

### ﻿*tRNA* and *rRNA* gene distribution

The positions of *tRNA* genes exhibited a remarkable degree of consistency across various species (Fig. 3; Suppl. material 1). However, certain black morels of *Morchellaimportuna* (Morimp1), *M.populiphila* (Morpop1), *M.punctipes* (Morpun1) and *M.tridentina* (Mortrid1), yellow morels of *Morchellafluvialis* (MorM1934m1), *M.steppicola* (Morpal1) and *M.diminutiva* (Mordim1) and blushing morel species of *M.anatolica* (Morana1) and *M.rufobrunnea* (Morruf1), along with false morels (*Morchellaceae*), display additional *tRNA* genes and rearrangements of the *tRNA* within their mitogenomes. These observed variations contributed to alterations in the relative order of the genes within the respective mitogenomes (Fig. 3; Suppl. material 1).

All the *Morchellaceae* species examined in this study contained *tRNA*s corresponding to all 20 natural amino acids within their mitogenomes. Notably, *trnR* (tRNA-Arg (ACG) and tRNA-Arg (TCT)) were observed with 4-7 copies, whereas *trnM* (tRNA-Met(CAT)), *trnS* (tRNA-Ser(TGA) and tRNA-Ser(GCT)), *trnL* (tRNA-Leu (TAA) and tRNA-Leu (TAG)) and *trnI* (tRNA-Ile (GAT) and tRNA-Ile (TAT)) exhibited 2-4 copies. The anticodons associated with *trnR*, *trnS*, *trnL* and *trnI* were CGU and AGA, UCA and AGC, UUA and CUA and AUC and AUA, respectively (Fig. 3; Suppl. material 1). Nevertheless, certain morel species, such as *M.brunnea* (Morbrun1), exhibited five copies of *trnC* (tRNA-Cys(GCA)), whereas three other morel species, namely *M.populiphila* (Morpop1), *M.diminutiva* (Mordim1) and *M.punctipes* (Morpun1), were characterised by only one copy of *trnI* (tRNA-Ile(GAT)) (Fig. 3; Suppl. material 1).

The positions of the *rRNA* genes within the *Morchellaceae* species in this study displayed a notable level of consistency, with *rnS* consistently appearing in close proximity to the *cox1* gene. However, certain morel species exhibit additional *rRNA* genes and *rRNA* rearrangements within their mitogenomes. For instance, *M.deliciosa* (Mordel1) has five *rnL* and three *rnS* genes, whereas *M.tridentina* (Mortrid1) and *M.ulmaria* (Morulm1) possess three *rnL* and two *rnS* genes. Furthermore, some species featured only one copy of the *rnL* or *rnS* gene or lacked it entirely, leading to distinct gene order variations (Fig. 3; Suppl. material 1).

### ﻿The distribution and phylogeny of HEGs within mitogenomes

A total of 925 LAGLIDADG (LAGs) and GIY-YIG (GIYs) genes and 913 introns have been identified within the mitogenomes of *Morchellaceae*. Individual species exhibited a range of 19–48 LAGs or GIYs. The presence and distribution of LAGs and GIYs were predominantly observed in PCGs, such as *cox1*, *cob*, *cox2* and *nad5* (Table 2; Suppl. material 7: figs S5, S6; Suppl. materials 1, 6). The number of LAGs with GIYs in the black morels was significantly higher than that in the yellow morels (Suppl. material 7: fig. S5; FDR adj. p < 0.05). Notably, GIY-YIG genes were limited to a small subset, identified in only five strains out of the total 30 fungi (Table 2).

**Figure 5.**
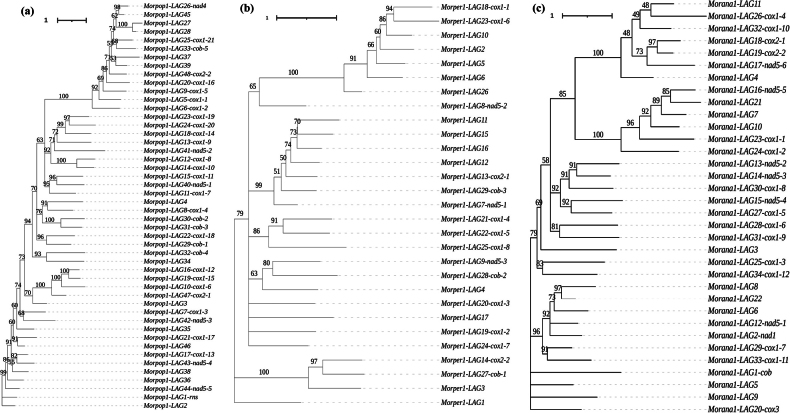
Phylogenetic trees of LAGLIDADG coding genes. Maximum-likelihood phylogenetic trees were constructed with three representative morel species based on the amino acid sequences of LAGLIDADG genes in their mitogenomes **a***Morchellapopuliphila* NRRL22315 (Morpop1), a black morel **b***Morchellaperuviana* NRRL66754 (Morper1), a yellow morel **c***Morchellaanatolica* PhC233 (Morana1), a blushing morel. Support values are shown at the nodes. Clades represent the amino acid sequences of all LAGLIDADG genes, which are either inserted within core genes or located externally in the mitogenomes. The genes are labelled with detailed information. For example, ”*Morpop1-LAG25-cox1-21*” describes the 25^th^ LAGLIDADG gene inserted into the core gene *cox1* at position 21 in *M.populiphila* NRRL22315, while, “*Morper1-LAG15*” denotes the 15^th^ LAGLIDADG gene located outside the core genes in *M.peruviana* NRRL66754.

**Figure 6.**
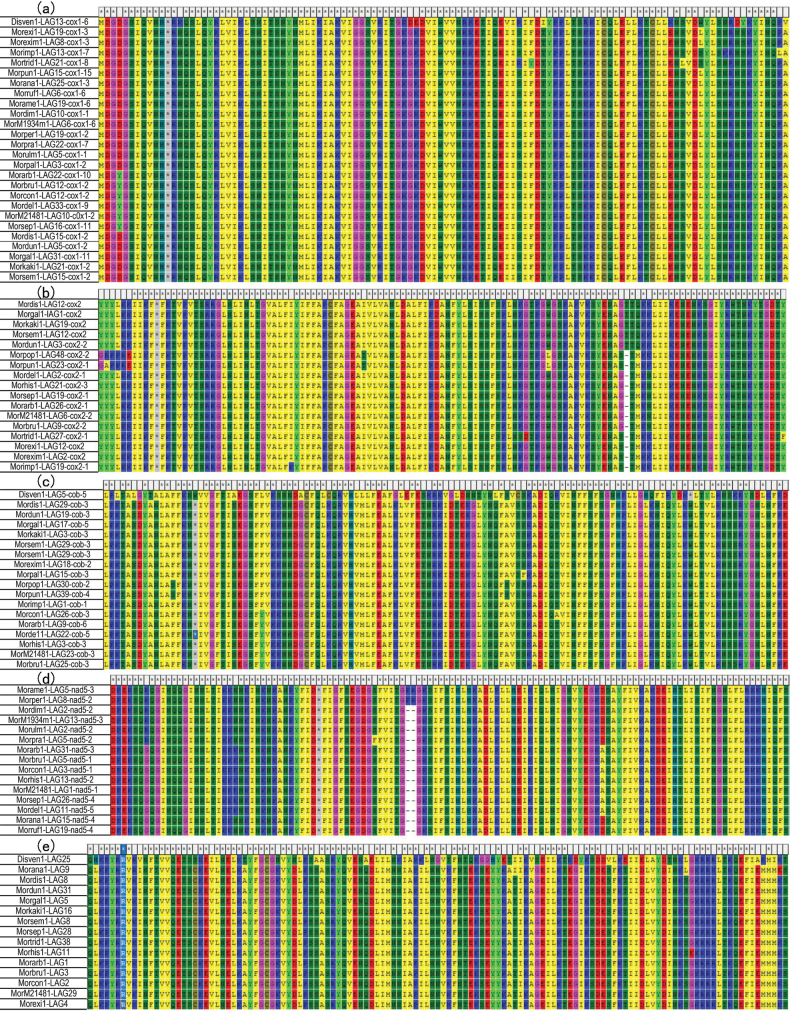
Alignments of LAGLIDADG amino acid sequences. Homologous amino acid sequences of LAGLIDADG coding genes were aligned across the *Morchella* species and false morels. These alignments encompass five representative LAGLIDADG genes present in the core genes; **a***cox1*, **b***cox2*, **c***cob*, **d***nad5*, and **e** outside the core genes. "*Morexi1-LAG19-cox1-3*" denotes the 19^th^ LAGLIDADG gene found at the position 3 of the core gene *cox1* in *Morchellaeximia* NRRL26621 (Morexi1).

The LAGs within each morel species displayed a significant diversity in size and content (Fig. 5). Phylogenetic trees segregated LAGs and GIYs of each representative morel into distinct clades with various motifs (Fig. 5). Examples include the black morel *M.populiphila* NRRL22315 (Morpop1) with a maximum of 48 LAGs, yellow morel *M.peruviana* NRRL66754 (Morper1) with 29 LAGs and blushing morel *M.anatolica* PhC233 (Morana1) with 34 LAGs (Table 2). The total number of LAGs with GIYs and those present in the *cox1* gene were highly associated with species relatedness (Suppl. material 7: fig. S6; R squared of PC1 and PC2 > 0.4; P < 0.05). Noteworthy instances include introns that house multiple LAGs. The total number of introns in the yellow and blushing morels was significantly higher than that in the black morels (Suppl. material 7: fig. S5; FDR adj. p < 0.05). A remarkable diversity of LAGs was evident in each morel mitogenome, with certain LAGs being identical or homologous across species (Fig. 6). Importantly, these LAGs were consistently inserted into core genes such as *cox1*, *cob*, *cox2* and *nad5*. For instance, within *cox1*, a singular subtype of LAG, Disven1-LAG13-cox1-6 (Fig. 6a), was shared between 25 morels and one false morel. This subtype exhibited a high degree of similarity, forming distinct clusters within the same or closely-related clades.

## ﻿Discussion

In this study, we sequenced the mitogenomes of species belonging to false, blushing, black and yellow morels of the *Morchellaceae* family. The assembly sizes for these mitogenomes varied from 217.7 kb to 565.1 kb, as shown in Table 1, with some species surpassing the size of the two previously-published morel mitogenomes (Liu et al. 2020a). The mitogenome of the yellow morel *M.crassipes* is 531.2 kb in size, whereas that of *M.importuna*, a black morel, is only 272.2 kb. It is important to note that the average mitogenome size within the *Morchella* genus is currently the largest known amongst fungal genera, with the mitogenome of *Morchellaprava*, a yellow morel, being the largest.

Mitochondrial genes, which are widely used in population genetics, evolution and phylogenetic analyses, present independent evolutionary characteristics that are distinct from those of nuclear genomes. The abundance of molecular markers, particularly mitochondria-specific genes, such as *atp6*, *atp8*, *atp9*, *cob*, *cox1*, *cox2*, *cox3*, *nad1*, *nad2*, *nad3*, *nad4*, *nad4L*, *nad5* and *nad6*, renders them valuable tools (Sanchez et al. 2018; Van de Paer et al. 2018; Wang et al. 2018). Combining datasets of mitochondrial genes, including those mentioned above, has proven to be a reliable molecular marker, demonstrating their potential as single-gene markers for evaluating the phylogenetic relationships of fungi (Korovesi et al. 2018; Li et al. 2019b).

Although *cox1* and other PCGs have not been extensively utilised as molecular markers in fungal phylogenetic analyses owing to limited mitochondrial genome availability, our study revealed identical and well-supported evolutionary tree topologies for *Morchellaceae* fungi, based on mitochondrial gene sets (Fig. 1). Notably, this phylogenetic analysis closely aligns with relationships derived from hundreds of conserved single-copy nuclear sequences, underscoring the robustness of our findings. In the context of fungal phylogenetic analysis, mitochondrial genes may emerge as an effective alternative classification method given their congruence with nuclear genomes. Mitochondria are postulated to have originated from the endosymbiosis of ancestral free-living Alphaproteobacteria, permanently integrated into the host cell (Munoz-Gomez et al. 2017). Currently, mitochondrial DNA (mtDNA) predominantly contains protein-coding genes crucial for oxidative phosphorylation and adenosine triphosphate (ATP) synthesis, serving as the primary energy production mechanism (Verma et al. 2018; Kulik et al. 2021). Additionally, mitochondria contribute to the production of metabolic precursors of macromolecules, such as proteins and lipids and generate metabolic by-products, such as ammonia and reactive oxygen species (Spinelli and Haigis 2018). These organelles are also pivotal for apoptosis, homeostasis and stress responses (Grahl et al. 2012; Verma et al. 2018; Zardoya 2020). Throughout evolution, many mitochondrial genes have migrated to the nuclear genome. In fungi, the majority of the genes associated with mitochondrial function are located in the nuclear genome (Bolender et al. 2008). For instance, in *Saccharomycescerevisiae*, the transfer of mtDNA to the nuclear genome occurs under specific nuclear gene mutations, depending on the mitogenome structure and sugar availability for fermentation, contributing to phenotypic variation in anaerobic environments (Shafer et al. 1999; Peter et al. 2018). Previous studies have demonstrated the absence of universally conserved genes in fungal mitogenomes, implying that the content of mitochondrial genes can vary considerably without compromising organelle function (Fonseca et al. 2021). This variability contributes to the adaptation of fungi to different environmental conditions (Malina et al. 2018). In this study, we observed substantial variation in the 15 PCGs amongst the major *Morchellaceae* clades, particularly in terms of size, GC content and AT and GC skews (Figs 2, 4). Although the order of the 15 PCGs remained highly conserved across most *Morchellaceae* species, the distribution of *rRNA* and*tRNA* genes exhibited additional rearrangements. These variations contributed to the relative order of the genes within the mitogenomes (Figs 1, 3). Our study further highlights the significant variability in core genes within the mitogenomes of different morel species. This variability may play a key role in the adaptation of diverse ecological groups, such as saprotrophic and mycorrhiza-like morels, throughout their evolutionary processes.

In Fungi, the length and genomic composition of mitogenomes may be influenced by the presence of accessory elements such as introns, HEGs and uORFs, introduced through horizontal gene transfer (Himmelstrand et al. 2014; Kanzi et al. 2016). In particular, the distribution of HEGs often reveals the extensive diversity observed in mitogenome size, genomic fragmentation and rearrangements. These endonucleases facilitate site-specific homologous recombination events, leading to the insertion, deletion, mutation or repair of DNA double-strand breaks. This phenomenon has been studied extensively in both yeast and mammalian cells (Rouet et al. 1994; Stoddard 2014).

Previous studies have demonstrated variations in the number and location of HEGs amongst the mitogenomes of six *Lactarius* species, which significantly contributes to differences in mitogenome organisation and size (Li et al. 2019b). Here, we explored the distribution and phylogeny of LAG and GIY HEG families. The abundance of LAGs and GIYs, ranging from 19 to 48, surpassed that observed for other fungi sequenced to date (Table 2; Suppl. material 7: fig. S5). LAGs displayed significant diversity in size and content within each species, with different *Morchellaceae* species sharing identical HEGs or homogeneous motifs.

For instance, Disven1-LAG13-cox1-6 was shared by 25 morels and one false-morel within *cox1* (Fig. 6a), indicating its ancestral nature. Conversely, some HEGs were shared by fewer morels, such as Disven1-LAG25, which was found in only 14 morels (Fig. 6e), suggesting a more recent evolutionary status. These findings imply strong evolutionary relationships between fungal LAGs and mitogenome size diversity, providing crucial evidence in support of the evolutionary theory proposed by Megarioti et al. (2020), which suggests that the co-evolution of introns and HEGs drives fungal mitogenome diversity (Megarioti et al. 2020).

The findings of this study also underscore the dynamic nature of *Morchella* mitogenomes and suggest a possible link between HEG abundance and evolutionary adaptability of these fungi (Fonseca et al. 2021). High copy numbers and shared motifs imply that HEGs may play roles beyond genome restructuring, potentially contributing to species-specific mitochondrial functions or adaptations to environmental niches (Belfort and Bonocora 2014; Fonseca et al. 2021).

A particularly intriguing, yet complex question pertains to the trophic status of morels. For the past two decades, it has been hypothesised that certain yellow morel species may form weak associations with trees (Y-mycorrhiza). However, this contention has not yet been substantiated by experimental studies. More broadly, it has been posited that yellow morels (Esculenta group) are associated with root systems during their life cycle, whereas black morels (Elata group) are exclusively saprotrophic. Our observation of a slightly larger genome size in yellow morels raises the question whether this difference is related to their ecology or biology. The observed variation in mitogenome size arose from differences in intron content, mobile genetic elements and repeat sequences (Tables 1, 2). These features may reflect evolutionary and ecological adaptation. For instance, intron proliferation and recombination events can lead to genome expansion or contraction (Tang et al. 2024). However, determining the direct effects of repeat elements on mitochondrial metabolism remains an unresolved question. Identifying correlations between the mitogenome structure (e.g., genome size, HEG copy number and GC%), gene order and ecological or lifestyle traits of morels is a compelling research avenue. To establish links between genetic characteristics and trophic status, extensive sequencing of morel mitogenomes and a comprehensive database of their biological and ecological traits are essential. Currently, knowledge of the biotrophic or saprotrophic nature of morels is limited to a few species, which impedes robust statistical analyses. Our study provides a resource for investigating these relationships; however, without a clear understanding of the trophic status of morel species, it is premature to propose a correlation between mitogenome size and trophic status.

Owing to their exceptional flavour, functional attributes and limited availability, the market value of wild morels has increased to approximately $300 per kilogram (dried) (Liu et al. 2017). Following Ower’s pioneering research in 1982, considerable progress has been made in morel cultivation in China since 2012, particularly for three black species, *M.importuna*, *M.sextelata* and *M.septimelata*, resulting in a cultivated area of 9,000 ha by 2018 (He et al. 2015; Liu et al. 2019). Despite these accomplishments, the morel-cultivation industry continues to grapple with persistent challenges that stem from unresolved issues. Notably, there is a dearth of documented successful cultivation instances for other yellow morels using either the recently proposed field cultivation model or the Ower’s indoor method (Liu et al. 2017). One of several factors leading to a decreased yield of morel-fruiting bodies in industrial cultivation regimes may be related to senescence of the mycelial inoculum and/or mutations in the nuclear and mitochondrial genomes. The expression of mitochondrial genes during morel cultivation needs to be investigated to understand the role of the mitogenome in fruiting body development (if any). Current mitogenomic resources can be used to unravel fundamental biological knowledge and other unidentified factors to improve the cultivation of black morels and the domestication of yellow morels.

## ﻿Conclusions

This study analysed the mitochondrial genomes of 30 *Morchellaceae* species, encompassing yellow, black, blushing and false morels, to elucidate their genetic architecture and ecological significance. The investigation revealed that these mitogenomes, ranging from 217 to 565 kbp in length, exist as either circular or linear DNA molecules with GC content between 38% and 48%. Although a substantial degree of gene order conservation was observed, several rearrangements were identified, most notably in *Morchellaimportuna*. This study also examined the diversity and phylogenetic relationships of HEGs within these mitogenomes, detecting between 19 and 48 HEGs per species. These observations support the contributions of introns and HEGs to mitogenomic diversity in morels. This research provides a novel perspective on the evolutionary dynamics of the *Morchellaceae* family.

## ﻿Additional information

### Adherence to national and international regulations

All the fungal strains used in this study have been legally obtained, respecting the Convention on Biological Diversity (Rio Convention).

### Author ORCIDs

Gang Tao https://orcid.org/0000-0002-0882-3752

Steven Ahrendt https://orcid.org/0000-0001-8492-4830

Shingo Miyauchi https://orcid.org/0000-0002-0620-5547

XiaoJie Zhu https://orcid.org/0009-0004-9333-5893

Hao Peng https://orcid.org/0009-0005-3348-1861

Kurt Labutti https://orcid.org/0000-0002-5838-1972

Alicia Clum https://orcid.org/0000-0002-5004-3362

Richard Hayes https://orcid.org/0000-0002-5236-7918

Patrick S. G. Chain https://orcid.org/0000-0003-3949-3634

Igor V. Grigoriev https://orcid.org/0000-0002-3136-8903

Gregory Bonito https://orcid.org/0000-0002-7262-8978

Francis M. Martin https://orcid.org/0000-0002-4737-3715

### Data availability

All of the data that support the findings of this study are available in the main text or Supplementary Information.

## Supplementary materials

Supplementary material 1 Comparisons of gene order in 30 mitochondrial genomes Data type xlsx https://binary.pensoft.net/file/1257965This dataset is made available under the Open Database License (http://opendatacommons.org/licenses/odbl/1.0/). The Open Database License (ODbL) is a license agreement intended to allow users to freely share, modify, and use this Dataset while maintaining this same freedom for others, provided that the original source and author(s) are credited.Gang Tao, Steven Ahrendt, Shingo Miyauchi, XiaoJie Zhu, Hao Peng, Kurt Labutti, Alicia Clum, Richard Hayes, Patrick S. G. Chain, Igor V. Grigoriev, Gregory Bonito, Francis M. Martin

Supplementary material 2 AT/GC content and skew Data type xlsx https://binary.pensoft.net/file/1257966This dataset is made available under the Open Database License (http://opendatacommons.org/licenses/odbl/1.0/). The Open Database License (ODbL) is a license agreement intended to allow users to freely share, modify, and use this Dataset while maintaining this same freedom for others, provided that the original source and author(s) are credited.Gang Tao, Steven Ahrendt, Shingo Miyauchi, XiaoJie Zhu, Hao Peng, Kurt Labutti, Alicia Clum, Richard Hayes, Patrick S. G. Chain, Igor V. Grigoriev, Gregory Bonito, Francis M. Martin

Supplementary material 3 Genomic coodinates of Morchellaceae mitogenomes Data type xlsx https://binary.pensoft.net/file/1257967This dataset is made available under the Open Database License (http://opendatacommons.org/licenses/odbl/1.0/). The Open Database License (ODbL) is a license agreement intended to allow users to freely share, modify, and use this Dataset while maintaining this same freedom for others, provided that the original source and author(s) are credited.Gang Tao, Steven Ahrendt, Shingo Miyauchi, XiaoJie Zhu, Hao Peng, Kurt Labutti, Alicia Clum, Richard Hayes, Patrick S. G. Chain, Igor V. Grigoriev, Gregory Bonito, Francis M. Martin

Supplementary material 4 Genomic statistics used for PERMANOVA Data type xlsx https://binary.pensoft.net/file/1257968This dataset is made available under the Open Database License (http://opendatacommons.org/licenses/odbl/1.0/). The Open Database License (ODbL) is a license agreement intended to allow users to freely share, modify, and use this Dataset while maintaining this same freedom for others, provided that the original source and author(s) are credited.Gang Tao, Steven Ahrendt, Shingo Miyauchi, XiaoJie Zhu, Hao Peng, Kurt Labutti, Alicia Clum, Richard Hayes, Patrick S. G. Chain, Igor V. Grigoriev, Gregory Bonito, Francis M. Martin

Supplementary material 5 Statistically significant variables in PERMANOVA Data type xlsx https://binary.pensoft.net/file/1257969This dataset is made available under the Open Database License (http://opendatacommons.org/licenses/odbl/1.0/). The Open Database License (ODbL) is a license agreement intended to allow users to freely share, modify, and use this Dataset while maintaining this same freedom for others, provided that the original source and author(s) are credited.Gang Tao, Steven Ahrendt, Shingo Miyauchi, XiaoJie Zhu, Hao Peng, Kurt Labutti, Alicia Clum, Richard Hayes, Patrick S. G. Chain, Igor V. Grigoriev, Gregory Bonito, Francis M. Martin

Supplementary material 6 Amino acid sequences of LAGs and GIYs Data type xlsx https://binary.pensoft.net/file/1257970This dataset is made available under the Open Database License (http://opendatacommons.org/licenses/odbl/1.0/). The Open Database License (ODbL) is a license agreement intended to allow users to freely share, modify, and use this Dataset while maintaining this same freedom for others, provided that the original source and author(s) are credited.Gang Tao, Steven Ahrendt, Shingo Miyauchi, XiaoJie Zhu, Hao Peng, Kurt Labutti, Alicia Clum, Richard Hayes, Patrick S. G. Chain, Igor V. Grigoriev, Gregory Bonito, Francis M. Martin

Supplementary material 7 Supplementary figures Data type pdf Explanation note **fig. S1.1–1.5**: Circular representation of the 30 *Morchella* mitogenomes; **fig. S2**: Linear representation of syntenic mitogenomes in black morels; **fig. S3**: Linear representation of syntenic mitogenomes in yellow and blushing morels; **fig. S4**: Phylogenetic trees of *atp6* and *atp8* genes; **fig. S5**: Trends of genomic features by fungal groups; **fig. S6**: Significant variables driving trends in 30 mitochondrial genomes; **fig. S7**: Correlated mitochondrial genes and genomes in size. https://binary.pensoft.net/file/1257971This dataset is made available under the Open Database License (http://opendatacommons.org/licenses/odbl/1.0/). The Open Database License (ODbL) is a license agreement intended to allow users to freely share, modify, and use this Dataset while maintaining this same freedom for others, provided that the original source and author(s) are credited.Gang Tao, Steven Ahrendt, Shingo Miyauchi, XiaoJie Zhu, Hao Peng, Kurt Labutti, Alicia Clum, Richard Hayes, Patrick S. G. Chain, Igor V. Grigoriev, Gregory Bonito, Francis M. Martin
