# Supplementary material for: ﻿Characterisation and comparative analysis of mitochondrial genomes of false, yellow, black and blushing morels provide insights on their structure and evolution
Source: IMA Fungus. 2025 Feb 21;16:e138363. doi: 10.3897/imafungus.16.138363 (PMC11881001; doi:10.3897/imafungus.16.138363)
Supplement: Supplementary material 7 — Supplementary figures [file imafungus-16-e138363-s007.pdf]

Supplementary Information

Characterization and comparative analysis of mitochondrial genomes of false, yellow, black and blushing morels provide insights on their structure and evolution

Gang Tao, Steven Ahrendt, Shingo Miyauchi, Xiaojie Zhu, Hao Peng, Kurt Labutti, Alicia Clum, Richard Hayes, Philippe Clowez, Greg Bonito, Igor V. Grigoriev, Francis M. Martin

List of Suppmpnentary Figure and Tables

Names	Descriptions
Fig. S1.1 - 1.5	Circular representation of the 30 Morchella mitogenomes.
Fig. S2	Linear representation of syntenic mitogenomes in black morels.
Fig. S3	Linear representation of syntenic mitogenomes in yellow and blushing morels.
Fig. S4	Phylogenetic trees of *atp6* and *atp8* genes.
Fig. S5	Trends of genomic features by fungal groups.
Fig. S6	Significant variables driving trends in 30 mitochondrial genomes.
Fig. S7	Correlated mitochondrial genes and genomes in size.
Table S1	Comparisons of gene order of mitogenomes
Table S2	AT/GC content and skew
Table S3	Genomic coodinates of Morchellaceae mitogenomes
Table S4	Genomic statistics used for PERMANOVA
Table S5	Statistically significant variables in PERMANOVA
Table S6	Amino acid sequences of LAGs and GIYs

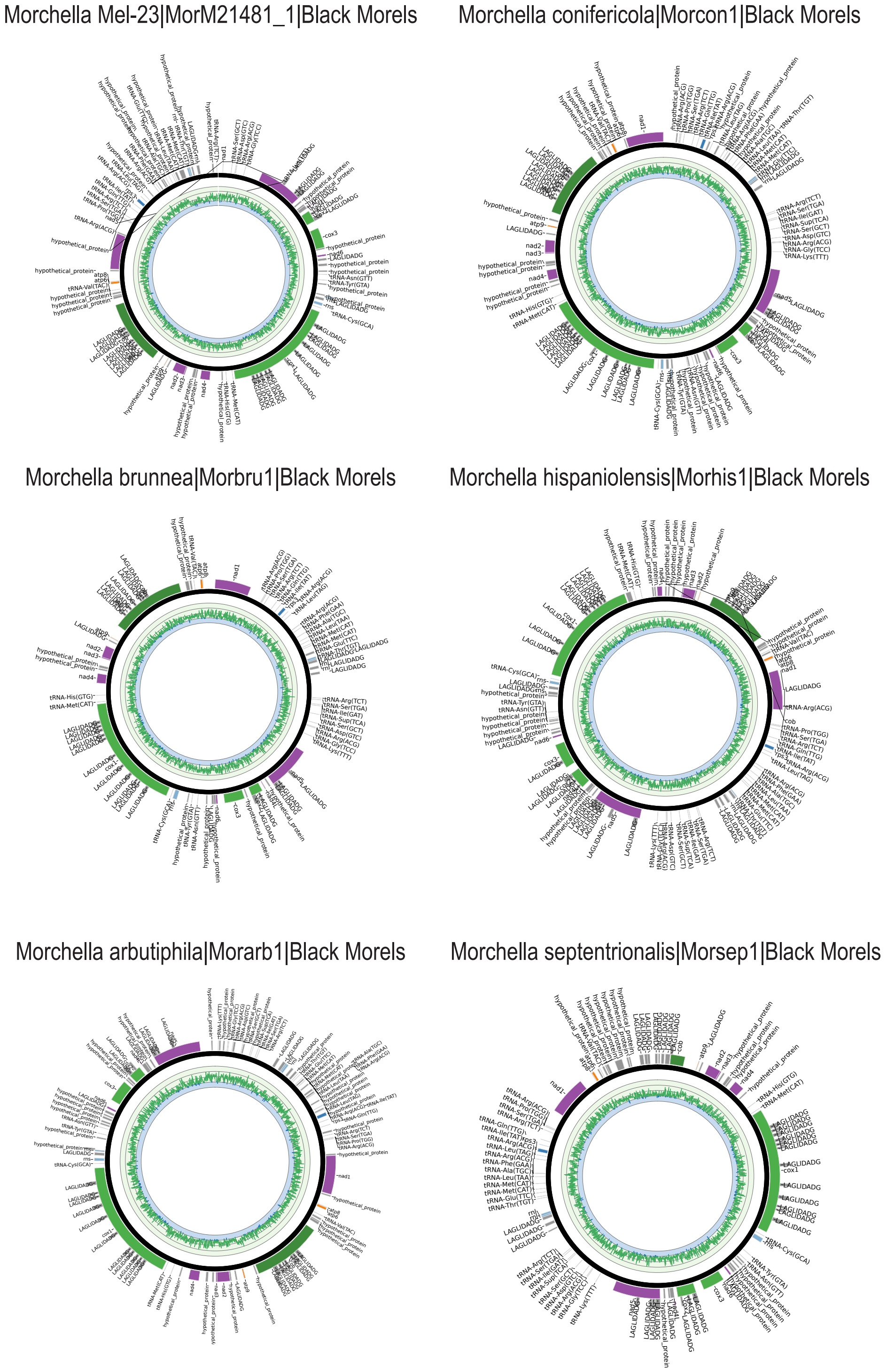

Figure S1.1. Circular representation of the 30 *Morchella* mitogenomes. Grey: Hypothetical/ homing endonuclease. Red: tRNA. Light blue: ribosomal genes (rnl, rns). Dark blue: rps3. The oxidative phosphorylation complexes are in various colours. Purple: Complex I (NADH:ubiquinone oxidoreductase), Dark green: Complex III (cytochrome bc1). Light green: Complex IV (cytochrome c oxidase). Orange: Complex V (ATP synthase). Blue lines linking points: Regions of the genome with >90% identity between them. Green wavy line: A sliding measure of GC%. The innermost line is 0%, and the outermost line is 100% with divisions every 25%.

Morchella sp. DIS|Mordis1|Black Morels

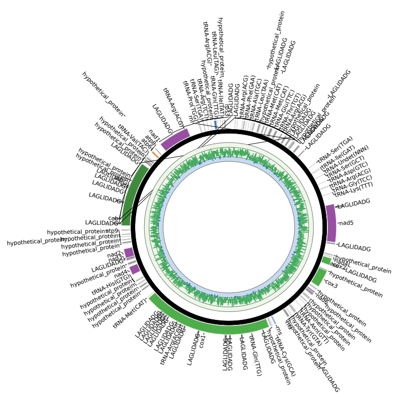

Morchella sp. SEM|Morsem1|Black Morels

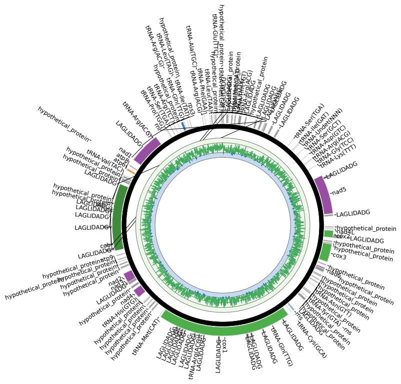

Morchella kakiicolor PhC280|Morkaki1|Black Morels

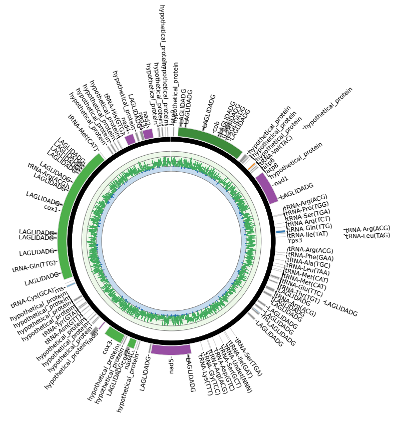

Morchella dunalii|Mordun1|Black Morels

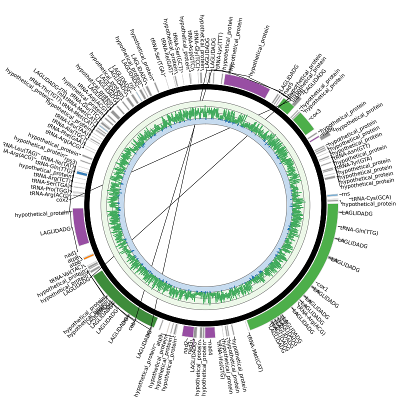

Morchella sp. GAL|Morgal1|Black Morels

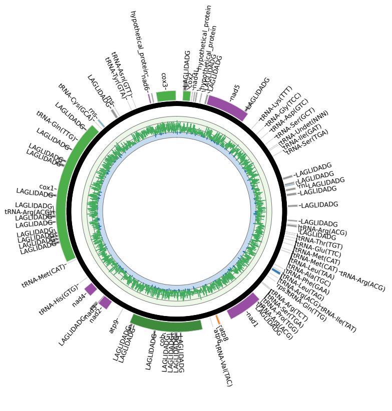

Morchella deliciosa|Mordel1|Black Morels

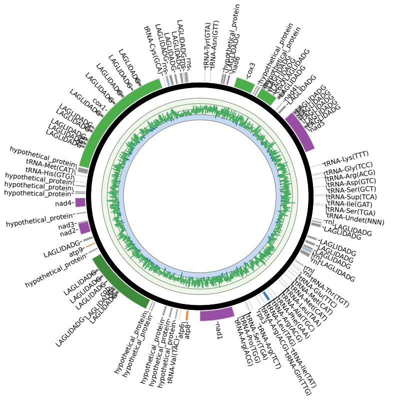

Figure S1.2. Circular representation of the 30 *Morchella* mitogenomes. Grey: Hypothetical/ homing endonuclease. Red: tRNA. Light blue: ribosomal genes (rnl, rns). Dark blue: rps3. The oxidative phosphorylation complexes are in various colours. Purple: Complex I (NADH:ubiquinone oxidoreductase), Dark green: Complex III (cytochrome bc1). Light green: Complex IV (cytochrome c oxidase). Orange: Complex V (ATP synthase). Blue lines linking points: Regions of the genome with >90% identity between them. Green wavy line: A sliding measure of GC%. The innermost line is 0%, and the outermost line is 100% with divisions every 25%.

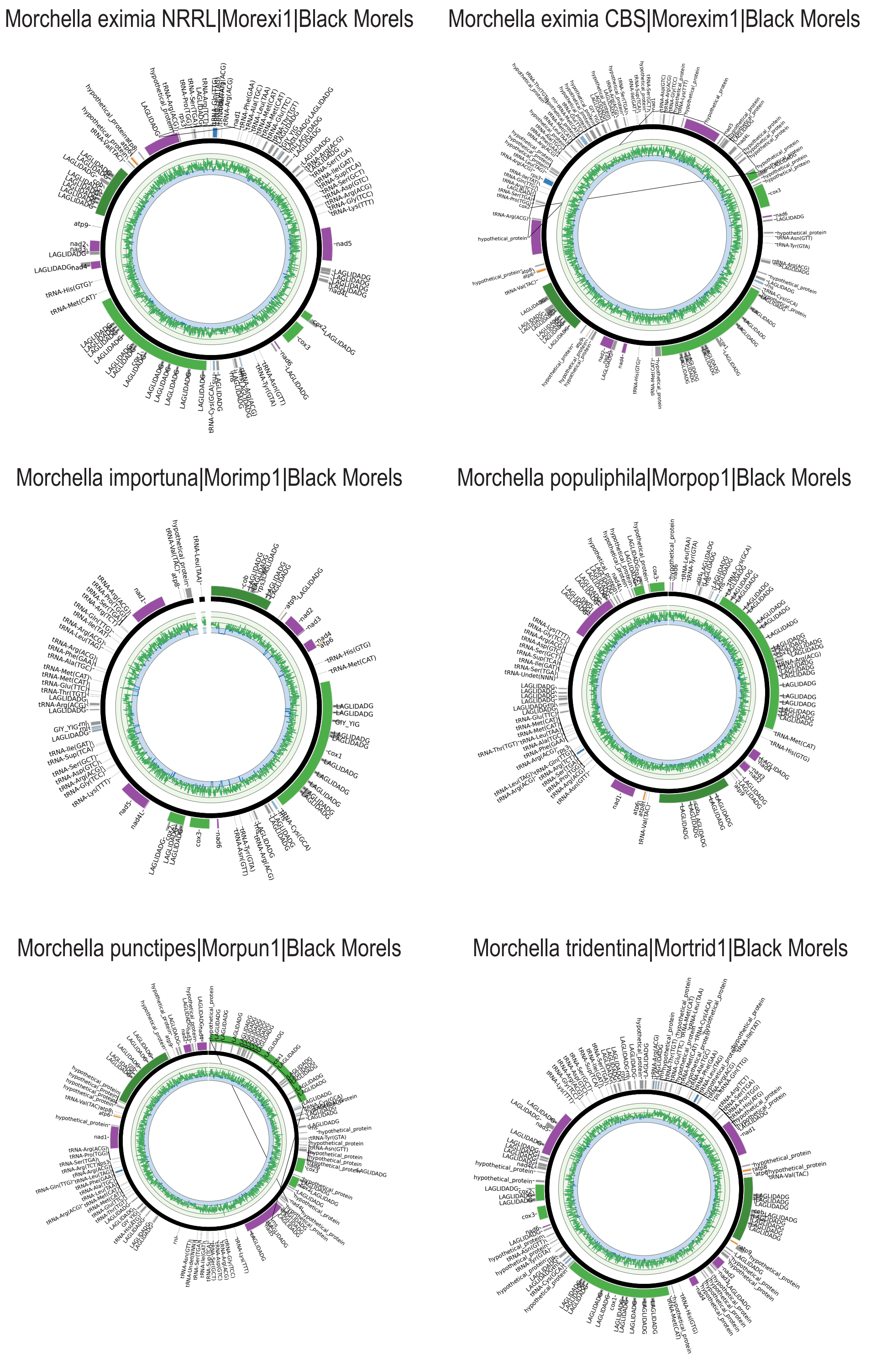

Figure S1.3. Circular representation of the 30 *Morchella* mitogenomes. Grey: Hypothetical/ homing endonuclease. Red: tRNA. Light blue: ribosomal genes (rnl, rns). Dark blue: rps3. The oxidative phosphorylation complexes are in various colours. Purple: Complex I (NADH:ubiquinone oxidoreductase), Dark green: Complex III (cytochrome bc1). Light green: Complex IV (cytochrome c oxidase). Orange: Complex V (ATP synthase). Blue lines linking points: Regions of the genome with >90% identity between them. Green wavy line: A sliding measure of GC%. The innermost line is 0%, and the outermost line is 100% with divisions every 25%.

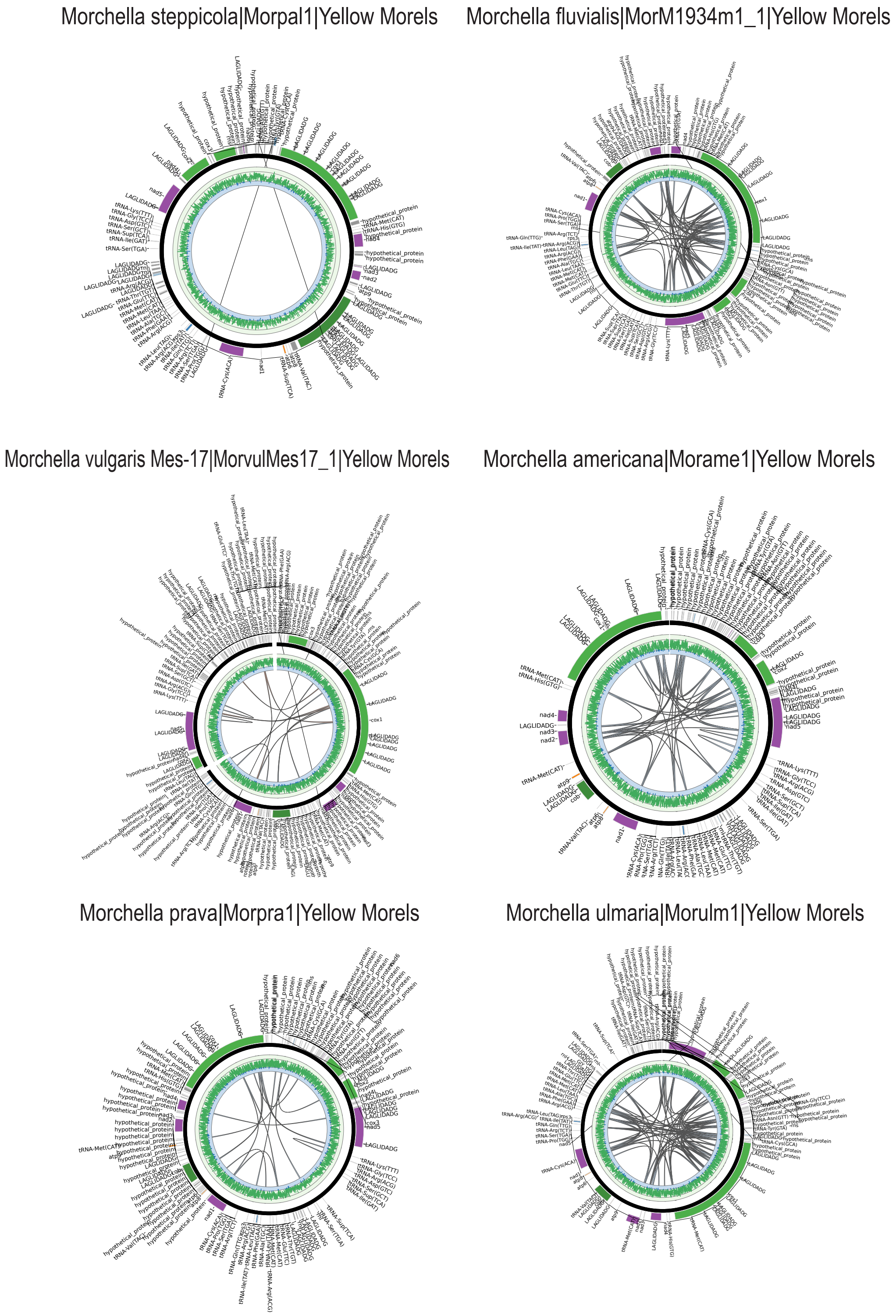

Figure S1.4. Circular representation of the 30 *Morchella* mitogenomes. Grey: Hypothetical/ homing endonuclease. Red: tRNA. Light blue: ribosomal genes (rnl, rns). Dark blue: rps3. The oxidative phosphorylation complexes are in various colours. Purple: Complex I (NADH:ubiquinone oxidoreductase), Dark green: Complex III (cytochrome bc1). Light green: Complex IV (cytochrome c oxidase). Orange: Complex V (ATP synthase). Blue lines linking points: Regions of the genome with >90% identity between them. Green wavy line: A sliding measure of GC%. The innermost line is 0%, and the outermost line is 100% with divisions every 25%.

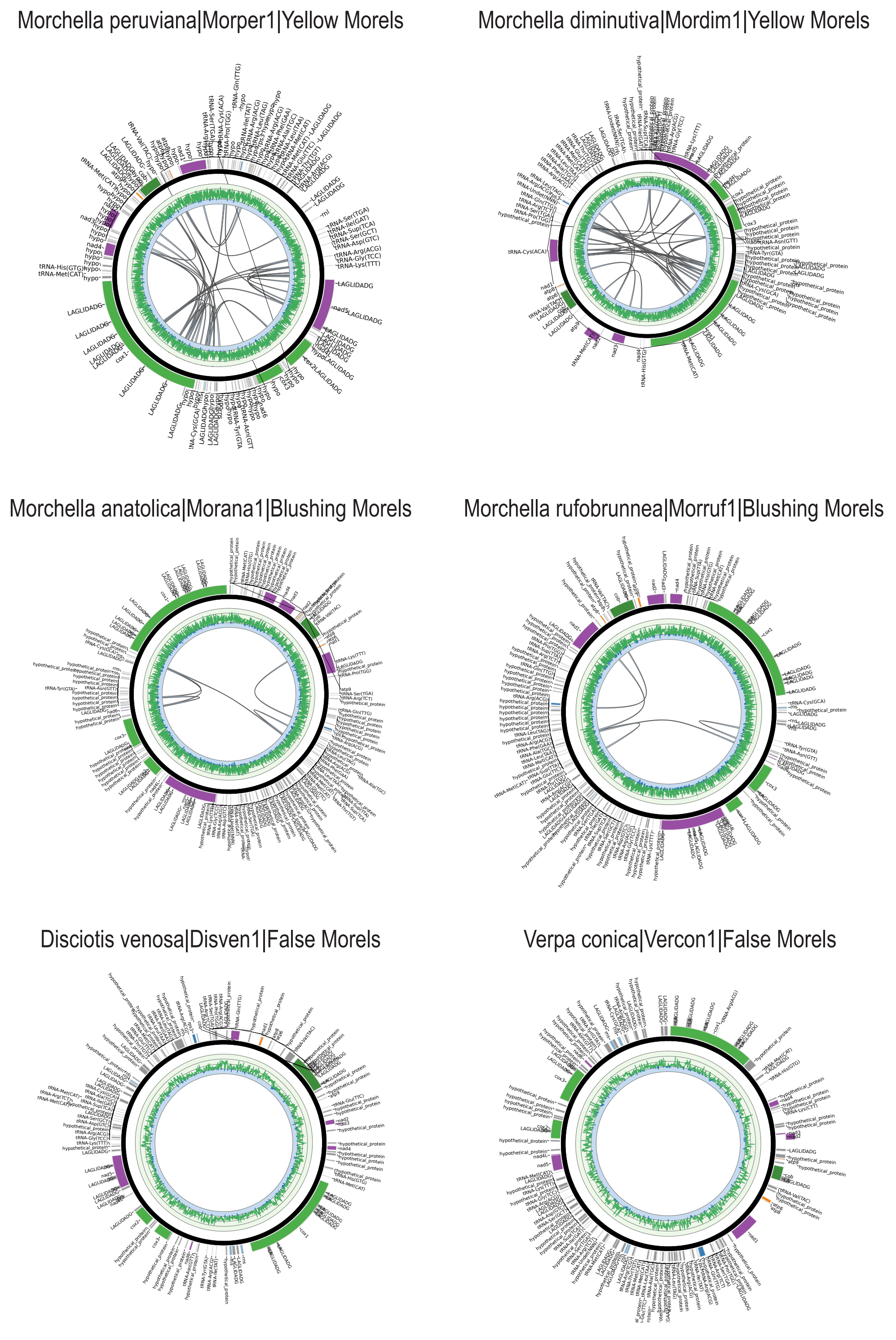

Figure S1.5. Circular representation of the 30 *Morchella* mitogenomes. Grey: Hypothetical/ homing endonuclease. Red: tRNA. Light blue: ribosomal genes (rnl, rns). Dark blue: rps3. The oxidative phosphorylation complexes are in various colours. Purple: Complex I (NADH:ubiquinone oxidoreductase), Dark green: Complex III (cytochrome bc1). Light green: Complex IV (cytochrome c oxidase). Orange: Complex V (ATP synthase). Blue lines linking points: Regions of the genome with >90% identity between them. Green wavy line: A sliding measure of GC%. The innermost line is 0%, and the outermost line is 100% with divisions every 25%.

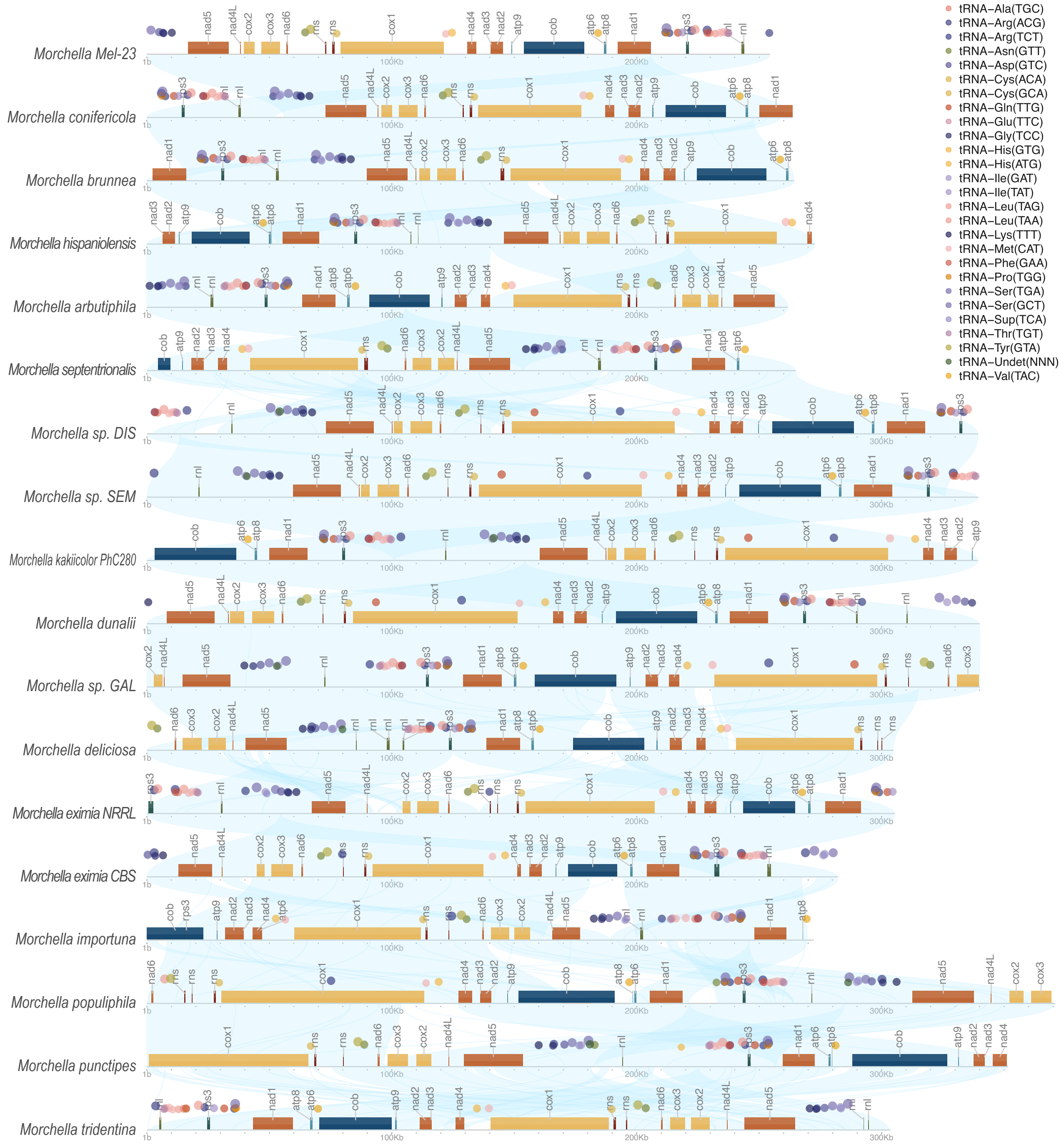

Figure S2. Linear representation of syntenic regions of mitogenomes. 18 black morels**.** The species are in the evolutionary order. Syntenic regions are connected by light-blue curves. Gray horizontal bars show the scaffold size and coordinates. Genes are illustrated as blocks with the gene names, and colors correspond to different gene types. Balloons represent sequences for *tRNA*. Each *tRNA* carrying different amino acids is color-coded. The balloon size and position correspond to the *tRNA* length. Genes coded in the positive and negative strands are merged into single bars. See the coordinates of the genes (Table S3). Note that the mitogenome of *Morchella* sp. Mes17 is fragmented.

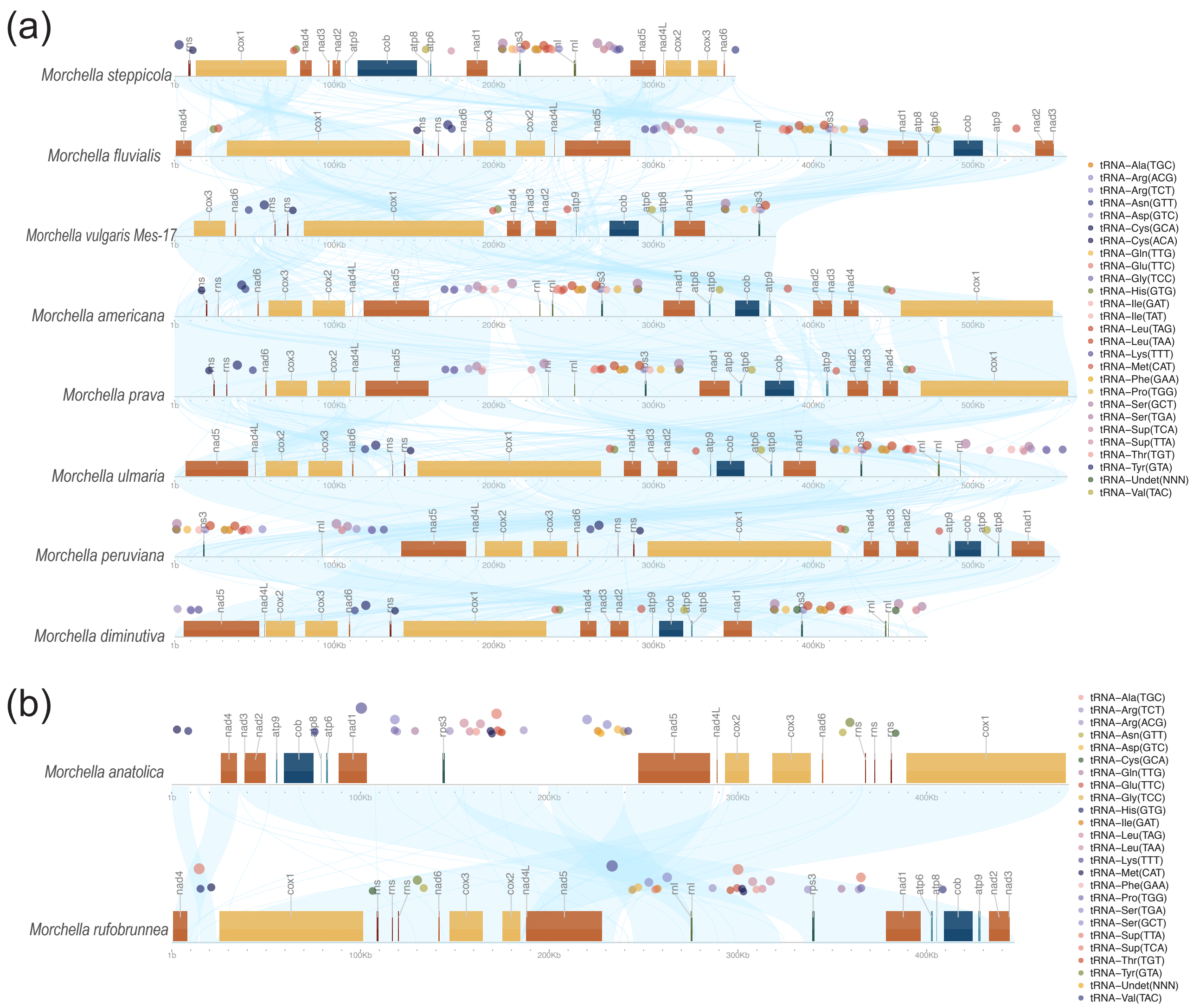

Figure S3. Linear representation of syntenic regions of mitogenomes. **(a)** Eight yellow morels**,** and **(b)** two blushing morels. The species are in the evolutionary order. Syntenic regions are connected by light-blue curves. Gray horizontal bars show the scaffold size and coordinates. Genes are illustrated as blocks with the gene names, and colors correspond to different gene types. Balloons represent sequences for *tRNA*. Each *tRNA* carrying different amino acids is color-coded. The balloon size and position correspond to the *tRNA* length. Genes coded in the positive and negative strands are merged into single bars. See the coordinates of the genes (Table S3). Note that the mitogenome of *Morchella* sp. Mes17 is fragmented.

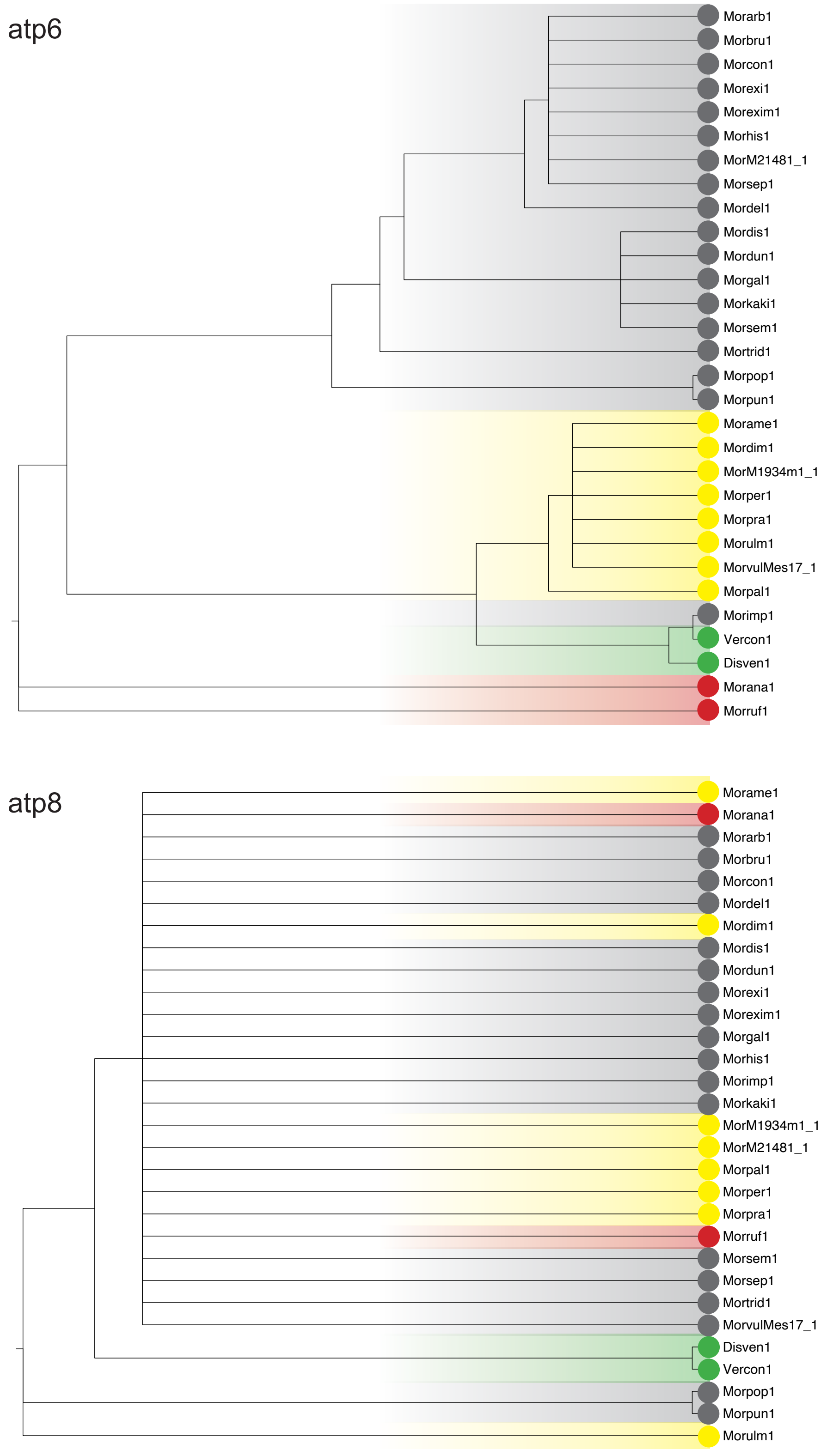

Figure S4. Phylogenetic trees of *atp6* and *atp8* genes. The trees were constructed with DNA sequences of the genes. The approximate maximum-likelihood method was used (see Methods). Phenotypic groups are colour-coded. Grey: Black morels. Yellow: Yellow morels. Red: Blushing morels. Green: False morels. The species are labeled with JGI fungal ID. See Table 1 for species names**.**

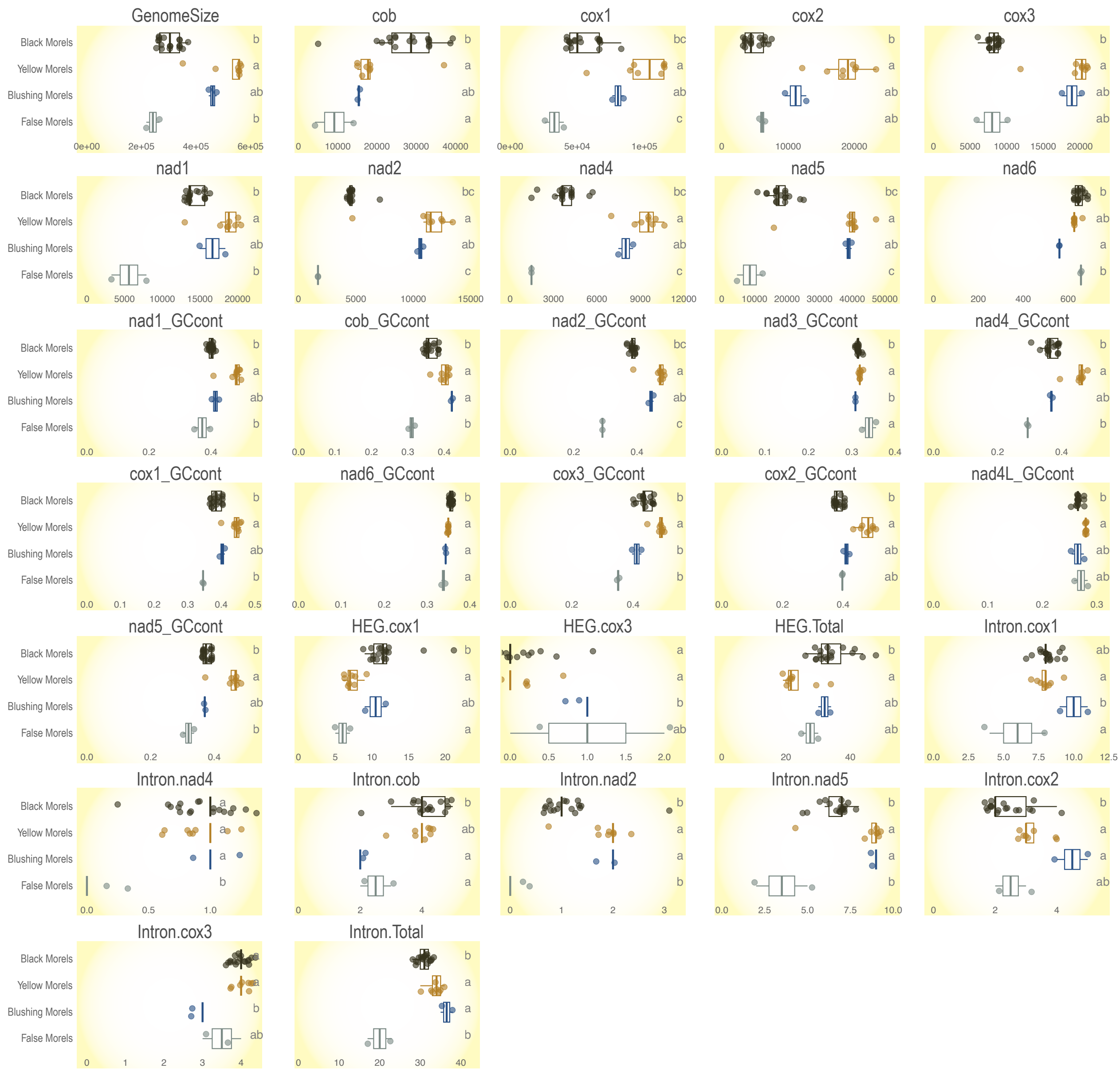

Figure S5. Trends of genomic features by fungal groups. The size of 13 core genes located in the mitogenomes. HEG: The number of homing endonuclease genes including LAGLIDADG and GIY-YIG. Intron: The count of introns. GCcont: GC content of the genes. The statistics of various genomic features is shown for the four ecological groups (black, yellow, blushing, and false morels). Alphabet letters indicate significant differences among the ecological groups (FDR adjusted $p < 0.05$; Kruskal-Wallis test with post hoc Dunn test). See Table S4 for details.

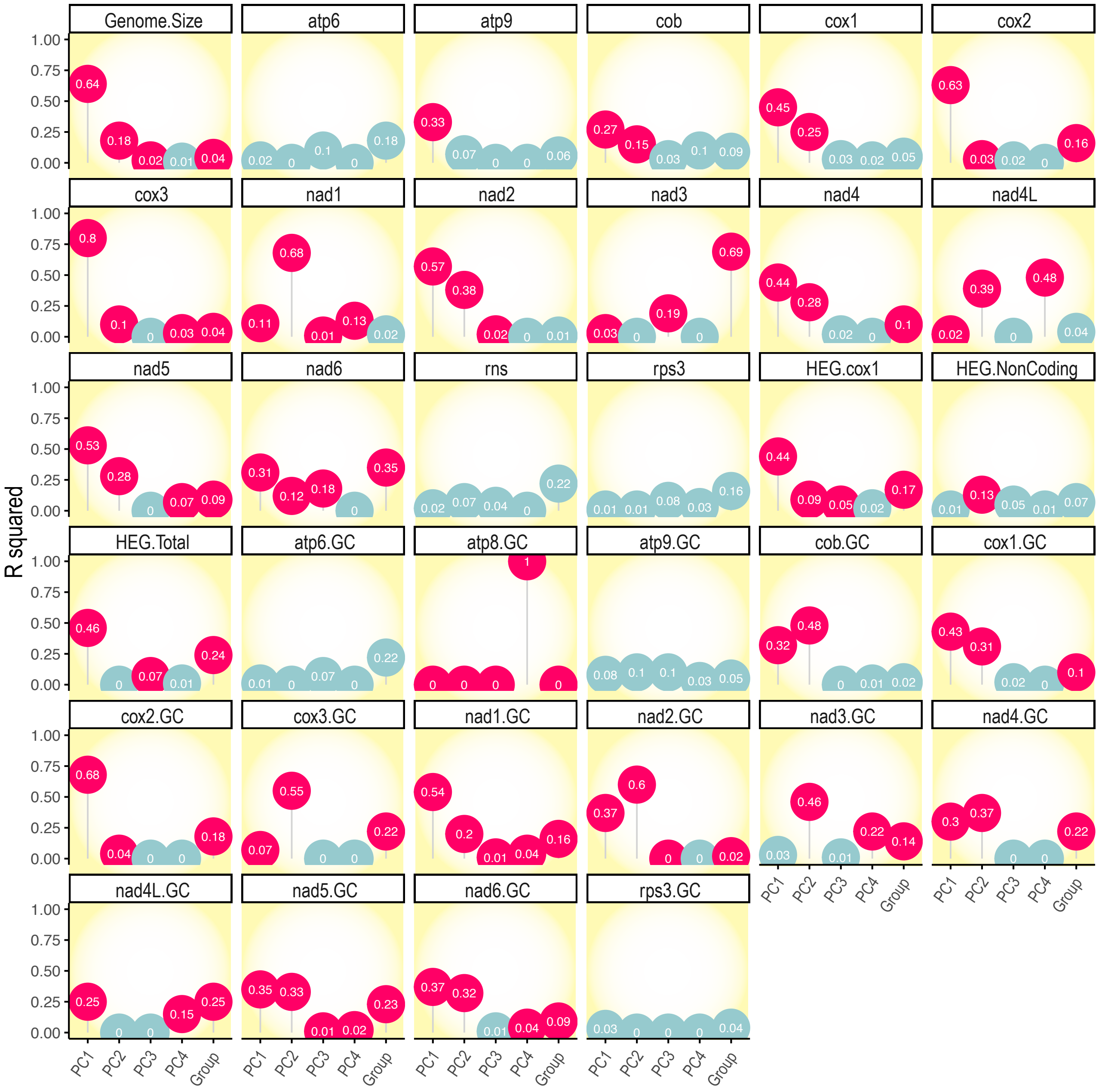

Figure S6. Significant variables driving trends in 30 mitochondrial genomes. The size of 15 core genes located in the mitogenomes. HEG: the number of homing endonuclease genes including LAGLIDADG and GIY-YIG. GC: GC content of the genes. Proportion of variation explained in selected genomic features. Circles with numbers inside indicating R squared values for variables tested. Circles in red indicate significant variables (p-value < 0.05; PERMANOVA model, Genomic feature ~ Phylogenetic distance + Fungal lifestyle). Major Principal component 1 to 4 explained over 90% (PC1:72.5%, PC2:11%, PC3:4.2%, PC4:3.4%) of variation in phylogenomic distances. Group: Fungal lifestyle. See Table S5.

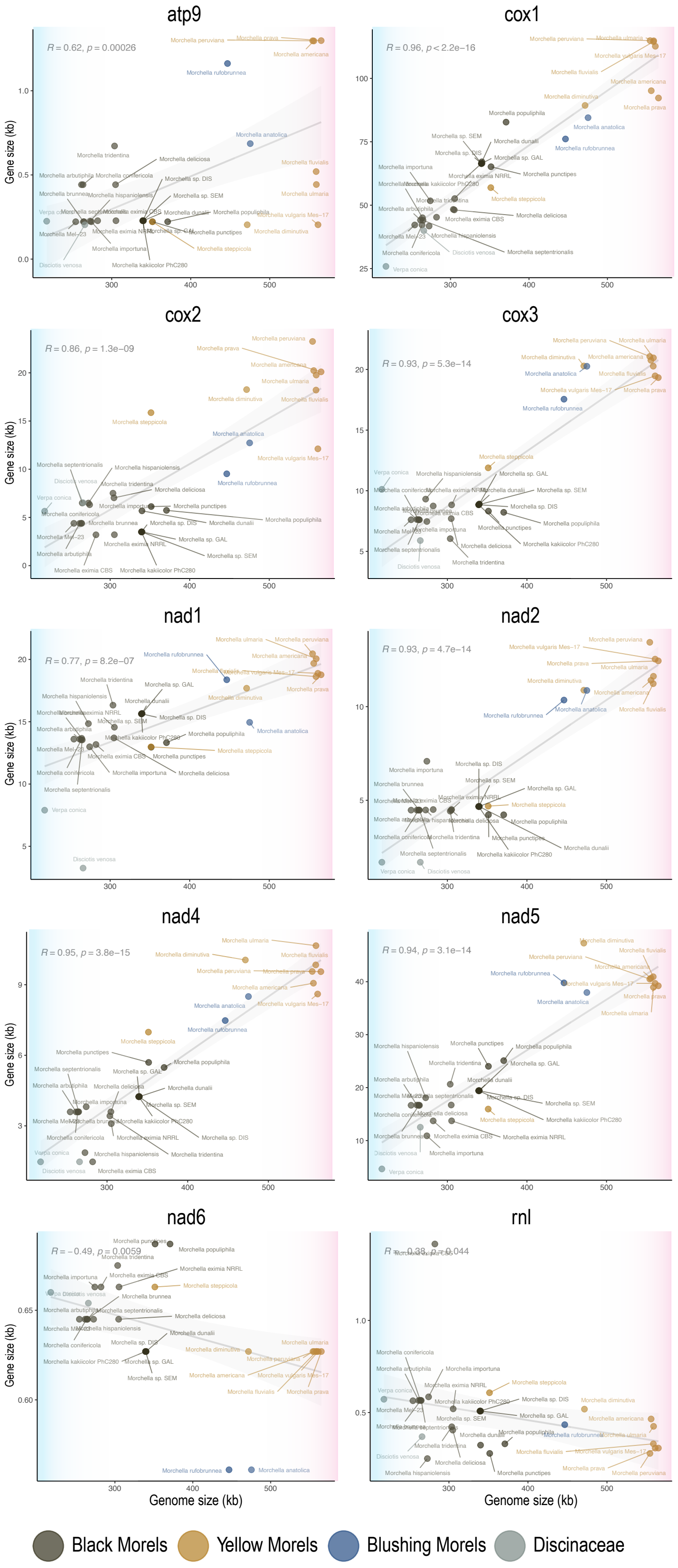

Figure S7. Correlated mitochondrial genes and genomes in size. The size of 30 genomes were plotted against the genes. Genes with statistical significance were shown (p > 0.05). The four fungal groups are colour-coded. Pearson coefficient (R) and p values were presented in the left top corner of the panels.
